# A Lightweight Anonymous Authentication Protocol with Perfect Forward Secrecy for Wireless Sensor Networks

**DOI:** 10.3390/s17112681

**Published:** 2017-11-21

**Authors:** Ling Xiong, Daiyuan Peng, Tu Peng, Hongbin Liang, Zhicai Liu

**Affiliations:** 1 School of Information Science and Technology, Southwest Jiaotong University, Chengdu 611756, China; lingdonghua99@163.com (L.X.); dypeng@swjtu.edu.cn (D.P.); 2 School of Software, Beijing Institute of Technology, Beijing 100081, China; pengtu@bit.edu.cn; 3 School of Transportation and Logistics, Southwest Jiaotong University, Chengdu 611756, China; 4 School of Computer and Software Engineering, Xihua University, Chengdu 610039, China; idle@gmail.com

**Keywords:** mutual authentication, user anonymity, wireless sensor networks, strand space model

## Abstract

Due to their frequent use in unattended and hostile deployment environments, the security in wireless sensor networks (WSNs) has attracted much interest in the past two decades. However, it remains a challenge to design a lightweight authentication protocol for WSNs because the designers are confronted with a series of desirable security requirements, e.g., user anonymity, perfect forward secrecy, resistance to de-synchronization attack. Recently, the authors presented two authentication schemes that attempt to provide user anonymity and to resist various known attacks. Unfortunately, in this work we shall show that user anonymity of the two schemes is achieved at the price of an impractical search operation—the gateway node may search for every possible value. Besides this defect, they are also prone to smart card loss attacks and have no provision for perfect forward secrecy. As our main contribution, a lightweight anonymous authentication scheme with perfect forward secrecy is designed, and what we believe the most interesting feature is that user anonymity, perfect forward secrecy, and resistance to de-synchronization attack can be achieved at the same time. As far as we know, it is extremely difficult to meet these security features simultaneously only using the lightweight operations, such as symmetric encryption/decryption and hash functions.

## 1. Introduction

**Figure 1:**
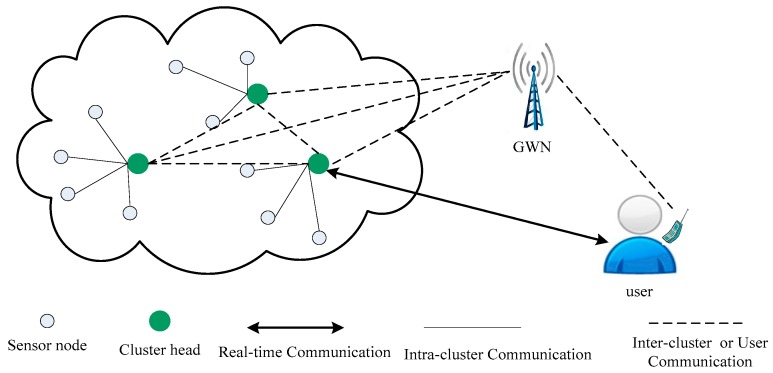
Real-time data access in WSNs.

Wireless sensor networks (WSNs) have gained a great deal of attention from researchers in the academic and industrial field mainly because of two reasons: first, they consist of a large number of resource-constrained sensor nodes, which are deployed randomly in a target region [1], and second, they can be widely used in various kinds of applications, such as healthcare monitoring [2], environment sensing [3], industrial monitoring [4], etc. Generally, WSNs are developed to monitor physical or environmental conditions, such as temperature, humidity, sound, etc. and collect real-time information about these conditions. In many applications [5,6,7], external users need to access to this real-time information from the sensor nodes. Figure 1 describes a way for real-time information access in WSNs. For example, using a WSN in the healthcare environment, the patient’s real-time information such as temperature, blood pressure, and pulse rate, will be collected by sensor nodes. Then, legitimate medical workers are able to access these data directly from the sensor nodes.

Although it seems appealing for users to access the real-time data from the sensor nodes, user authentication has been a critical issue in WSNs due to their frequent use in unattended and hostile environments [8]. Because many applications for WSNs operate in such environments, such as battlefields, a malicious adversary could easily control the communication channel, i.e., he/she would be able to eavesdrop, insert, block, and alter the transmitted data. Thus, WSNs are subject to various types of attacks. To ensure that only authorized users can access the reliable sensor nodes and to protect the real-time information, it is indispensable to achieve mutual authentication and establish a session key between the user and the sensor node. Nowadays, there are mainly three ways to accomplish authenticated key establishment scheme in WSNs [9].

- The first and the simplest solution for the authenticated key establishment is a shared symmetric key between the user and the sensor node. In this case, if a WSN has *n* sensor nodes and *m* users, each sensor node needs to store *m* symmetric keys, each user needs to store *n* symmetric keys, and the WSN needs to establish *nm* symmetric keys.
- Secondly, using public key cryptography, like ECC [10], RSA [11] or ElGamal [12], is another approach to complete authenticated key establishment.
- Third, the user and the sensor node can achieve mutual authentication and establish a session key through a trust gateway node (GWN) [13,14,15,16,17,18,19,20,21,22,23,24,25,26,27,28,29,30]. In this case, both the user and the sensor node need to share only a single key with the GWN. The GWN can help the user and the sensor authenticate each other and distribute a shared secret session key at each session. After this phase, the user can use this session key to access the real-time data from the desired sensor node without involving the GWN.

Obviously, the first method does not scale well, and the second way using public key cryptography primitives may tend to be resource intensive because most of them are based on the large integer. Hence, the authenticated key establishment scheme with the help of the GWN is even more admired owing to limited computation and communication resources, capability, bandwidth of sensor nodes. Additionally, identity masquerade and identity tracing have become common attacks in WSNs, which will cause the problem of identity privacy. Hence, there is a growing demand to achieve anonymous authentication in WSNs. Besides, since the sensor node is unattended, the long-term key of the sensor node may be compromised by an adversary. In this case, the previous session keys will be in danger. To address it, perfect forward secrecy should be considered. Therefore, anonymous authentication schemes with perfect forward secrecy for WSNs should be designed by using only the lightweight cryptographic primitives, such as symmetric key encryption/decryption and hash functions.

Many anonymous authentication schemes using lightweight cryptographic primitive have been proposed for WSNs in the past several years. However, as far as we know, most of them cannot consider perfect forward secrecy or suffer from de-synchronization attack. In this work, we design a lightweight authentication scheme for WSNs, which can achieve user anonymity, perfect forward secrecy, and resistance to de-synchronization attack at the same time.

### 1.1. Related Works

In some applications for WSNs, such as real-time healthcare monitoring, traffic control monitoring, and military surveillance, external users are interested in accessing real-time data directly from desired sensor nodes without involving the GWN. User authentication is an essential security measure for the user to be first authorized to the GWN as well as the sensor nodes before granting access to the real-time data. To achieve user authentication in WSNs, hundreds of schemes have been proposed in the last decade, such as remarkable schemes [13,14,15,16,17,18,19,20,21,22,23,24,25,26,27,28,29,30]. In 2006, Wong et al. [13] designed a dynamic strong-password authentication scheme for WSNs using lightweight operations, such as one-way hash function and XOR operations. But later, Das [14] pointed out that Wong et al.’s scheme is vulnerable to replay attack and stolen-verifier attack. In order to address these issues, Das [14] presented a two-factor authenticated key establishment scheme for WSNs, which claimed to provide strong authentication and resist various kinds of attacks. Unfortunately, a series of articles [15,16,17,18,19] have indicated that Das’s scheme [14] has still some drawbacks and flaws, such as susceptibility to privileged-insider attacks, smart card loss attacks, and parallel session attacks.

Although the abovementioned schemes [15,16,17,18,19] have much better performance than Das’ scheme [14], they are still prone to several security flaws, such as smart card loss attacks and forgery attacks. In 2012, Das et al. [20] developed a better scheme to solve these weaknesses. However, the security of Das et al.’s new scheme was not satisfactory, because of its vulnerability to some attacks [21,22,23]. After that, Xue et al. [24] designed a temporal-credential based authenticated key agreement scheme for WSNs using the hash function and XOR operations, which claimed to provide identity and password protection, and resiliency of smart card loss attacks. Unfortunately, Jiang et al. [25] described how the Xue et al.’s scheme [24] was insecure against identity guessing attack, privileged insider attack, tracking attack and smart card loss attack. They proposed an efficient two-factor user authentication scheme with unlinkability property in WSNs. The unlinkability pseudonym identity can help the GWN to quickly search the exact communicating user. Besides, it is able to provide user anonymity and resistance to smart card loss attack. In 2016, Das [26] proposed an enhanced authentication scheme based on Jiang et al.’s scheme [25]. He insisted that their scheme can provide higher security level than other schemes.

To the designers’ disappointment, both Jiang et al.’s scheme and Das’ scheme have been found vulnerable to the desynchronization attack [31]. Most recently, Gope and Hwang [27] proposed a realistic authentication scheme for WSNs, which can ensure various kinds of imperative security properties like mutual authentication, user anonymity, perfect forward secrecy, etc. For the communication between the user and the GWN, Gope and Hwang’s scheme employed a set of unlinkable shadow-IDs and emergency keys to prevent de-synchronization attack [31]. This is the preferred way to solve the de-synchronization attack. However, for the communication between the GWN and the sensor node, if the adversary blocks the response message flow from the sensor node, the communication will be lost in synchronization. Thus the sensor node needs to ask the GWN for the new secret shared key. Besides, we observe that their scheme also cannot resist against known session-specific temporary information attack [28]. When the session-specific temporary information *N_u_* is disclosed to the adversary, it is obvious that *K_ug_* can be resumed from transmitted message *N_x_* = *N_u_*⊕*K_ug_*. Then, the adversary generates his own Ts*_ugnew_ and the session key SK*, and computes Ts* = *h*(*K_ug_*||*ID_U_*||*N_u_*)⊕Ts*_ugnew_, SK*’’ = *h*(*K_ug_*||*ID_U_*||*N_u_*)⊕SK*, *V*_4_ = *h*(SK*’’||*N_u_*||Ts*||*K_ug_*), where *ID_U_* is the identity of the user, which can be off-line guessed by transmitted messages *Ks_ug_* and *AID_U_* = *h*(*ID_U_*||*K_ug_*||*N_u_*||*Ks_ug_*). Thus, the adversary can successfully forge a legal gateway node authentication message and get the session key. 

In the same year, the authors of [28,29] proposed a lightweight authentication scheme, which uses a ‘dynamic ID technique’ to achieve user anonymity and is secure in resisting known session-specific temporary information attack. Unfortunately, we find that the two schemes are insecure against smart card loss attacks. Besides, both schemes have two design flaws, including impractical GWN search operation and no provision for perfect forward secrecy.

On the other hand, perfect forward secrecy is an important security property for authenticated schemes. Unfortunately, to the best of our knowledge, most of the authentication schemes only using lightweight cryptographic primitives cannot provide perfect forward secrecy (e.g., the recent pertinent authentication schemes [24,25,26,28,29,30]). Although Mir et al. [30] claimed that their scheme is secure in perfect forward secrecy, we find out it is still prone to forward secrecy attack. In this scheme, the authors proved that the session key *SK* = *h*(*K_i_*||*K_j_*||*ID_i_*||*SID_i_*||*T*_1_) is secure under the assumption that the adversary *A* does not obtain the identity *ID_i_* of the user. However, if GWN’s secret key *d* is compromised, *A* can offline guess *ID_i_* through the transmitted message *M* as below. *A* guesses a candidate *ID*’*_i_* and computes *X*’*_i_* = *h*(*ID*’*_i_*||*d*), *ID_i_*||*K_i_*||*T*_1_||*H_i_* = *D_X’i_*(*M*). *A* checks whether *ID*’*_i_* and *ID_i_* are equivalent. If they are equal, *A* can obtain the correct *ID_i_*. Otherwise, *A* repeats this operation until the correct *ID_i_* is obtained. Hence, Mir et al.’s scheme is unable to achieve perfect forward secrecy. Several articles [32,33,34] pointed out that it is intrinsically unable to provide perfect forward secrecy in the scheme that does not employ public-key primitives. To the best of our knowledge, some schemes [27] tried to address this issue using the one-time hash chain technique. However, it may cause de-synchronization attack because the hash chain value will be updated after each successful session. 

### 1.2. Motivation and Contributions

Two previously-thought sound schemes [28,29] use ‘dynamic ID technique’ to achieve user anonymity at the price of the impractical exhaustive search operations. The reason is that users’ real identities are encoded into dynamic identities, no one is able to get the identity information of the user without the secret key. When the user wants to access the WSNs systems, it is difficult for the GWN to tell apart the real identity of the user. As a result, the GWN needs to search for every possible parameter to figure out the exact user. Generally, to address this, a pseudonym identity method [25,26] is used to help the GWN to read the correct information from the user information table. In this way, both of the user and the GWN store a randomly generated pseudonym identity, which is updated after each successful session. Since the pseudonym identity is different at each session, the adversary cannot track a specific user. However, Wang et al. [31] pointed out the scheme using pseudonym identity may easily suffer from the de-synchronization attacks, which may render the scheme completely unusable unless the user or the sensor node re-registers. 

To the best of our knowledge, the hash chain technique can be employed to ensure perfect forward secrecy for lightweight cryptographic protocols [27]. However, like the pseudonym identity method, both communicating parties need to update their shared one-time hash chain value after completion of each session. Thus, the technique may also cause the de-synchronization attack.

Motivated by the above facts, we construct a new efficient authentication scheme for WSNs using the pseudonym identity method and one-time hash chain technique to achieve user anonymity and perfect forward secrecy. For the communication between the user and the GWN, the back-end of GWN stores two pseudonym identities *PID_i_*_0_ and *PID_i_*_1_ to resist against de-synchronization attack. *PID_i_*_0_ stores the value of the new pseudonym identity. *PID_i_*_1_ has two functions: the one is storing the value of the old pseudonym identity, the other is a tag for updating hash chain. If *PID_i_*_1_ = ⊥, it means that the value of hash chain has updated in the previous session. Otherwise, the value of hash chain does not change, where ⊥ denotes null. For the communication between the GWN and the sensor node, serial number technique is used to resist against de-synchronization attack.

Altogether, in this paper, we analyze the security of two representative schemes [28,29] for WSNs and show their vulnerability to smart card loss attack, impractical GWN search operation and no provision for perfect forward secrecy. To overcome these weaknesses, we design a lightweight anonymous authentication protocol for WSNs based on the one-time hash chain and pseudonym identity. The main contributions of our scheme are summarized as follows:

(1) The proposed scheme is resilient to various kinds of known attacks, such as de-synchronization attack, known session-specific temporary information attack;
(2) The proposed scheme can provide mutual authentication, user anonymity, and perfect forward secrecy, etc.
(3) The proposed scheme uses lightweight cryptographic primitives, such as symmetric encryption/decryption and hash functions. It is very suitable for the resource constrained sensor nodes.

### 1.3. Adversary Model

An adversary *A* has five goals. The first is that *A* can successfully impersonate the user *U_i_* authenticating to GWN. The second is that *A* can successfully impersonate GWN authenticating to *U_i_*. The third is that *A* can successfully impersonate the sensor node *S_nj_* authenticating to GWN. The fourth is that *A* can successfully impersonate GWN authenticating to *S_nj_*. And the last is that *A* can obtain the session key among *U_i_*, GWN and *S_nj_*. We assume that *A* is a probabilistic polynomial time attacker, and the feasible attacks are summarized as follows:

➢ *A* can control the channel among *U_i_*, GWN and *S_nj_*. It means that *A* can eavesdrop, insert, block, and alter the transmitted messages through the public communication channel.
➢ *A* can obtain one of the two authentication factors, smart card or password. If *A* has obtained the smart card, he can extract the secret value in the smart card and has the capability of enumerating identity and password space |D_ID_*D_PW_|.
➢ *A* may be another legitimate but malicious user in the system.
➢ *A* may be a legitimate but malicious sensor node.

### 1.4. Notations

All the notations mentioned in two related schemes and our proposed scheme are defined in Table 1.

### 1.5. Organization of the Paper

This paper takes two relate schemes [28,29] as case studies, we present a concrete attack to show that the two schemes are insecure against the smart card loss attack. Besides, we also show both schemes have two design flaws, including impractical GWN search operation and no provision for perfect forward secrecy. Then, we put forward a new way to deal with de-synchronization attack and design an efficient anonymous authentication scheme with perfect forward secrecy for WSNs. 

The rest of this paper is organized as follows: Section 2 reviews two related schemes for WSNs. Section 3 presents the detailed procedure of the proposed scheme. Section 4 gives security analysis of our scheme. The computation and communication costs analysis of the proposed scheme are discussed in Section 5. Finally, Section 6 concludes this paper.

## 2. Review of Two Related Schemes

This section will describe two related authenticated key establishment schemes for WSNs, which are Lu et al.’s scheme [28] and Jung et al.’s scheme [29]. The reason for choosing these two schemes is that they are the typical representations of recent schemes in WSNs which have the security flaws in smart card loss attack, impractical GWN search operation and no provision for perfect forward secrecy. First, we will give briefly review of two schemes. Later on, the detailed weaknesses of the two schemes will be described. 

### 2.1. Review of Lu et al.’s Scheme

**Figure 2:**
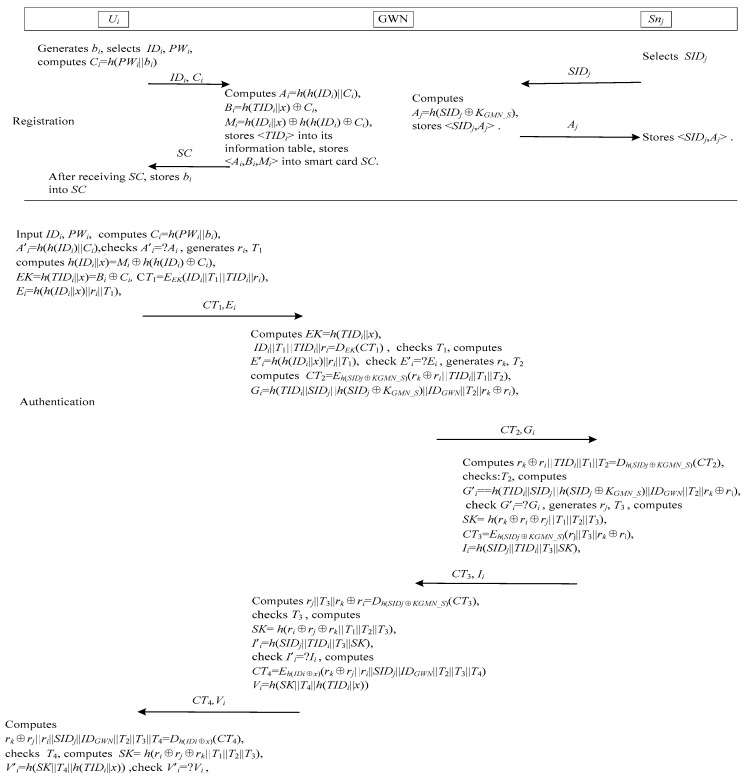
The authentication and key agreement phase of Lu et al.’s scheme.

Lu et al.’s authentication scheme [28] is shown in Figure 2. This scheme consists of four phases: the user registration, the sensor node registration, login and authentication, password change.

#### 2.1.1. User Registration

*Step 1*: A new user *U_i_* selects the identity *ID_i_* and the password *PW_i_*, generates a random number *b_i_*. Then *U_i_* computes *C_i_* = *h*(*PW_i_*||*b_i_*), *U_i_* transmits {*ID_i_*,*C_i_*} to GWN through a secure channel.

*Step 2*: Upon receipt of the message, the GWN computes *A_i_* = *h*(*h*(*ID_i_*)||*C_i_*), *B_i_* = *h*(*TID_i_*||*x*)⊕*C_i_*, *M_i_* = *h*(*ID_i_*||*x*)⊕*h*(*h*(*ID_i_*)⊕*C_i_*). After that, GWN stores {*TID_i_*} in its memory, and stores {*A_i_*,*B_i_*,*M_i_*} into smart card *SC*. Finally, GWN sends *SC* to *U_i_* via a private channel.

*Step 3*: After receiving *SC* from GWN, *U_i_* stores *b_i_* into *SC*.

#### 2.1.2. Sensor Node Registration

*Step 1*: A new sensor node *S_nj_* selects identity *SID_j_* and transmits {*SID_j_*} to GWN through a secure channel.

*Step 2*: The GWN computes *A_j_* = *h*(*SID_j_*⊕*K_GMN_S_*), and returen it to *S_nj_* after storing {*SID_j_*,*A_j_*} into its memory.

*Step 3*: After receiving *SID_j_*, *A_j_* from GWN, *S_nj_* stores them into its memory as the secret.

#### 2.1.3. Login

When a user *U_i_* desires the WSNs services, he/she needs to achieve mutual authenticate with GWN and *S_nj_*. As shown in Figure 2, the process of mutual authentication is described as follows.

*Step 1*: *U_i_* inputs *ID_i_* and *PW_i_* into the smart card *SC*. *SC* computes *C_i_* = *h*(*PW_i_*||*b_i_*), *A*’*_i_* = *h*(*h*(*ID_i_*)||*C_i_*)*,* and compares *A*’*_i_* with the stored value *A_i_*. If they are not equal, *SC* terminates the session. Otherwise, *SC* believes *U_i_* as a legitimate user. Next, *SC* generates a random number *r_i_*, and computes *h*(*ID_i_*||*x*) = *M_i_*⊕*h*(*h*(*ID_i_*)⊕*C_i_*), *EK* = *h*(*TID_i_*||*x*) = *B_i_*⊕*C_i_,* C*T*_1_ = *E_EK_*(*ID_i_*||*T*_1_||*TID_i_*||*r_i_*), *E_i_* = *h*(*h*(*ID_i_*||*x*)||*r_i_*||*T*_1_), where *T*_1_ is the timestamp. Finally, *SC* sends the login request {*CT*_1_*,E_i_*} to GWN through the public channel.

*Step 2*: After receiving the login messages, the GWN computes *EK* = *h*_1_(*TID_i_*||*x*), *ID_i_*||*T*_1_||*TID_i_*||*r_i_* = *D_EK_*(C*T*_1_). Then, the GWN checks the timestamp *T*_1_, computes *E*’*_i_* = *h*(*h*(*ID_i_*||*x*)||*r_i_*||*T*_1_), and checks whether *E*’*_i_* matches with the received *E_i_*. If it does not hold, GWN terminates the session. Otherwise, the GWN generates a random numnber *r_k_*, and computes *CT*_2_ = *E_h_*_(*SIDj*__⊕*KGMN_S*)_(*r_k_*⊕*r_i_*||*TID_i_*||*T*_1_||*T*_2_), *G_i_* = *h*(*TID_i_*||*SID_j_*||*h*(*SID_j_*⊕*K_GMN_S_*)||*ID_GWN_*||*T*_2_||*r_k_*⊕*r_i_*), where *T*_2_ is the timestamp. Finally, the GWN sends {*CT*_2_,*G_i_*} to the sensor node *S_nj_* that *U_i_* wants to interact with via the public channel. 

*Step 3*: Upon receiving the messages {*CT*_2_,*G_i_*} from GWM, *S_nj_* at first computes *r_k_*⊕*r_i_*||*TID_i_*||*T*_1_||*T*_2_ = *D_h_*_(*SIDj*__⊕*KGMN_S*)_(*CT*_2_). Then, *S_nj_* checks the timestamp *T*_2_, computes *G*’*_i_* = *h*(*TID_i_*||*SID_j_*||*h*(*SID_j_*⊕*K_GMN_S_*)||*ID_GWN_*||*T*_2_||*r_k_*⊕*r_i_*), and checks whether *G*’*_i_* matches with the received *G_i_*. If it does not hold, *S_nj_* terminates the session. Otherwise, the *S_nj_* generates a random numnber *r_j_*, and computes *SK* = *h*(*r_k_*⊕*r_i_*⊕*r_j_*||*T*_1_||*T*_2_||*T*_3_), *CT*_3_ = *E_h_*_(*SIDj*__⊕*KGMN_S*)_(*r*_j_||*T*_3_||*r_k_*⊕*r*_i_), *I_i_* = *h*(*SID_j_*||*TID_i_*||*T*_3_||*SK*), where *T*_3_ is the timestamp. Finally, *S_nj_* transmits {*CT*_3_, *I_i_*} to GWN.

*Step 4*: GWN first computes *r_j_*||*T*_3_||*r_k_*⊕*r_i_* = *D_h_*_(*SIDj*__⊕*KGMN_S*)_(*CT*_3_). Then, the GWN checks the timestamp *T*_3_, and computes *SK* = *h*(*r_k_*⊕*r_i_*⊕*r_j_*||*T*_1_||*T*_2_||*T*_3_), *I*’*_i_* = *h*(*SID_j_*||*TID_i_*||*T*_3_||*SK*), and checks whether *I*’*_i_* matches with the received *I_i_*. If it does not hold, GWN terminates the session. Otherwise, the GWN computes *CT*_4_ = *E_h_*_(*IDi*__⊕*x*)_(*r_k_*⊕*r_j_*||*r_i_*||*SID_j_*||*ID_GWN_*||*T*_2_||*T*_3_||*T*_4_), *V_i_* = *h*(*SK*||*T*_4_||*h*(*TID_i_*||*x*)), where *T*_4_ is the timestamp. Finally, GWN transmits {*CT*_4_, *V_i_*} to *U_i_*. 

*Step 5*: *U_i_* computes *r_k_*⊕*r_j_*||*r_i_*||*SID_j_*||*ID_GWN_*||*T*_2_||*T*_3_||*T*_4_ = *D_h_*_(*IDi*__⊕*x*)_(*CT*_4_), and checks the timestamp *T*_4_. Then *U_i_* computes *SK* = *h*(*r_k_*⊕*r_i_*⊕*r_j_*||*T*_1_||*T*_2_||*T*_3_), *V*’*_i_* = *h*(*SK*||*T*_4_||*h*(*TID_i_*||*x*)), and checks whether *V*’*_i_* matches with the received *V_i_*. If it holds, *U_i_* completes the authentication. Otherwise, *U_i_* fails to authenticate the GWN. 

#### 2.1.4. Password Update Phase

When a user *U_i_* wants to update the password, he/she needs to execute the following steps:

*Step 1*: *U_i_* inputs *ID_i_*, *PW_i_* into the smart card *SC*. *SC* computes *C_i_* = *h*(*PW_i_*||*b_i_*), *h*(*TID_i_*||*x*) = *B_i_*⊕*C_i_, h*(*ID_i_*||*x*) = *M_i_*⊕*h*(*h*(*ID_i_*)⊕*C_i_*), *A*’*_i_* = *h*(*h*(*ID_i_*)||*C_i_*), and checks whether *A*’*_i_* and *A_i_* are equal. If not, *SC* fails to authenticate *U_i_*, and rejects the request of the password update. Otherwise *U_i_* inputs a new password PW*_i_. 

*Step 2*: SC computes C*_i_ = h_0_(PW*_i_||b_i_), B*_i_ = h(TID_i_||x)⊕C_i_⊕C*_i_, M*_i_ = h(ID_i_||x)⊕h(h(ID_i_)⊕C*_i_) and A*_i_ = h(h(ID_i_)||C*_i_).

*Step 3*: Finally, A*_i_,B*_i_, and M*_i_ are stored in *SC* to replace *A_i_*, *B_i_*, and *M_i_* respectively.

### 2.2. Review of Jung et al.’s Scheme

**Figure 3:**
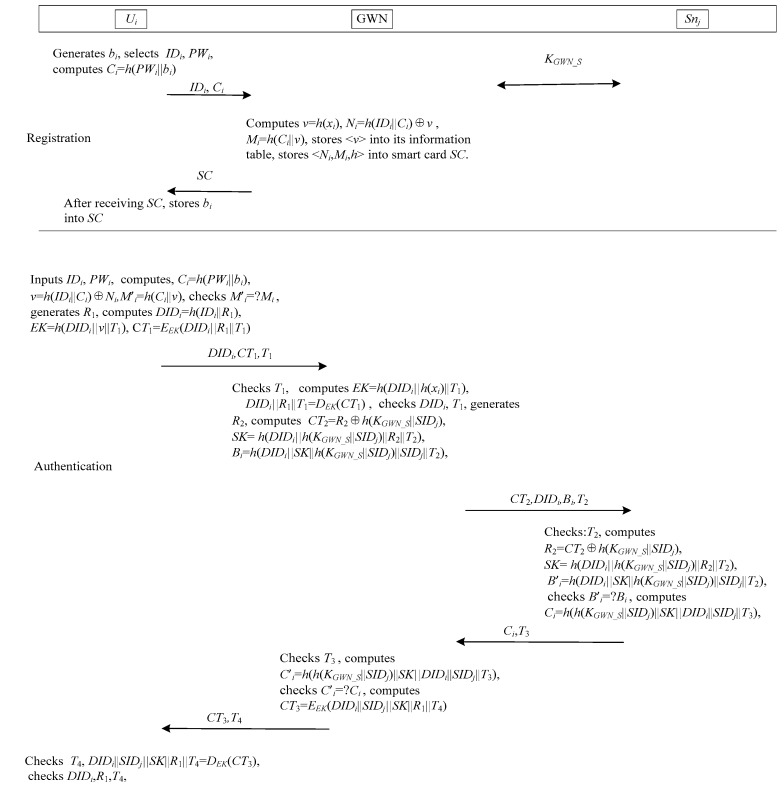
The authentication and key agreement phase of Jung et al.’s scheme.

Jung et al.’s authentication scheme [29] is shown in Figure 3. This scheme consists of three phases: registration, login and authentication, password change. This scheme has not sensor node registration phase. When the sensor node is developed, a shared key *K_GWN_S_* between the sensor node and the GWN is assigned. 

#### 2.2.1. User Registration

*Step 1*: A new user *U_i_* selects the identity *ID_i_* and the password *PW_i_*, generates a random number *b_i_*. Then *U_i_* computes *C_i_* = *h*(*PW_i_*||*b_i_*), *U_i_* transmits {*ID_i_*,*C_i_*} to GWN through a secure channel.

*Step 2*: Upon receipt of the message, the GWN computes *v* = *h*(*x_i_*), *N_i_* = *h*(*ID_i_*||*C_i_*)⊕*v*, *M_i_* = *h*(*C_i_*||*v*). After that, GWN stores {*v*} in its memory, and stores {*N_i_*,*M_i_*,*h*} into smart card *SC*. Finally, GWN sends *SC* to *U_i_* via a private channel.

*Step 3*: After receiving *SC* from GWN, *U_i_* stores *b_i_* into *SC*.

#### 2.2.2. Login and Authentication

When a user *U_i_* desires the WSNs services, he/she needs to achieve mutual authenticate with GWN and *S_nj_*. Figure 3 illustrates the process of mutual authentication for the proposed scheme. In detail, the process is:

*Step 1*: *U_i_* inputs *ID_i_* and *PW_i_* into the smart card *SC*. *SC* computes *C_i_* = *h*(*PW_i_*||*b_i_*), *v* = *h*(*ID_i_*||*C_i_*)⊕*N_i_, M*’*_i_* = *h*(*C_i_*||*v*)*,* and compares *M*’*_i_* with the stored value *M_i_*. If they are not equal, *SC* terminates the session. Otherwise, *SC* believes *U_i_* as a legitimate user. Next, *SC* generates a random number *R*_1_, and computes *DID_i_* = *h*(*ID_i_*||*R*_1_), *EK* = *h*(*DID_i_*||*v*||*T*_1_), C*T*_1_ = *E_EK_*(*DID_i_*||*R*_1_||*T*_1_), where *T*_1_ is the timestamp. Finally, *SC* sends the login request {*DID_i_*, *CT*_1_*,* *T*_1_} to GWN through the public channel.

*Step 2*: After receiving the login messages, the GWN first checks the timestamp *T*_1_, and computes *EK* = *h*(*DID_i_*||*h*(*x_i_*)||*T*_1_), *DID_i_*||*R*_1_||*T*_1_ = *D_EK_*(*CT*_1_). Then, the GWN checks whether *DID_i_* and *T*_1_ matches with the received values. If they do not hold, GWN terminates the session. Otherwise, the GWN generates a random numnber *R*_2_, and computes *CT*_2_ = *R*_2_⊕*h*(*x_S_*||*SID_j_*), *SK* = *h*(*DID_i_*||*h*(*K_GWN_S_*||*SID_j_*)||*R*_2_||*T*_2_), *B_i_* = *h*(*DID_i_*||*SK*||*h*(*K_GWN_S_*||*SID_j_*)||*SID_j_*||*T*_2_), where *T*_2_ is the timestamp. Finally, the GWN sends {*CT*_2_,*DID_i_*,*B_i_*,*T*_2_} to the sensor node *S_nj_*. 

*Step 3*: Upon receiving the messages {*CT*_2_,*DID_i_*,*B_i_*,*T*_2*i*_} from GWM, *S_nj_* first checks the timestamp *T*_2_, and computes *R*_2_ = *CT*_2_⊕*h*(*K_GWN_S_*||*SID_j_*), *SK* = *h*(*DID_i_*||*h*(*K_GWN_S_*||*SID_j_*)||*R*_2_||*T*_2_), *B*’*_i_* = *h*(*DID_i_*||*SK*||*h*(*K_GWN_S_*||*SID_j_*)||*SID_j_*||*T*_2_). Then, the *S_nj_* checks whether *B*’*_i_* matches with the received *B_i_*. If it does not hold, *S_nj_* terminates the session. Otherwise, the *S_nj_* computes *C_i_* = *h*(*h*(*K_GWN_S_*||*SID_j_*)||*SK*||*DID_i_*||*SID_j_*||*T*_3_), where *T*_3_ is the timestamp. Finally, *S_nj_* transmits {*C_i_*,*T*_3_} to GWN.

*Step 4*: GWN first checks the timestamp *T*_3_, and computes *C*’*_i_* = *h*(*h*(*K_GWN_S_*||*SID_j_*)||*SK*||*DID_i_*||*SID_j_*||*T*_3_). Then, the GWN checks whether *C*’*_i_* matches with the received *C_i_*. If it does not hold, GWN terminates the session. Otherwise, the GWN computes *CT*_3_ = *E_EK_*(*DID_i_*||*SID_j_*||*SK*||*R*_1_||*T*_4_), where *T*_4_ is the timestamp. Finally, GWN transmits {*CT*_3_, *T*_4_} to *U_i_*. 

*Step 5*: *U_i_* checks the timestamp *T*_4_ and computes *DID_i_*||*SID_j_*||*SK*||*R*_1_||*T*_4_ = *D_EK_*(*CT*_3_). Then *U_i_* checks whether *DID_i_*, *R*_1_, and *T*_4_ matches with the previous values. If it holds, *U_i_* completes the authentication. Otherwise, *U_i_* fails to authenticate the GWN.

#### 2.2.3. Password Update Phase

When a user *U_i_* wants to update the password, he/she needs to execute the following steps:

*Step 1*: *U_i_* inputs *ID_i_*, *PW_i_* into the smart card *SC*. *SC* computes *C_i_* = *h*(*PW_i_*||*b_i_*), *v* = *h*(*ID_i_*||*C_i_*)⊕*N_i_*, *M*’*_i_* = *h*(*C_i_*||*v*), and checks whether *M*’*_i_* and the stored *M_i_* are equal. If not, *SC* fails to authenticate *U_i_*, and rejects the request for the password update. Otherwise *U_i_* inputs a new password PW*_i_. 

*Step 2*: SC computes C*_i_ = h_0_(PW*_i_||b_i_), N*_i_ = v⊕h(ID_i_||C*_i_), and M*_i_ = h(C*_i_||v).

*Step 3*: Finally, N*_i_ and M*_i_ are stored in *SC* to replace *N_i_* and *M_i_* respectively. 

### 2.3. Security Analysis of Two Related Schemes

The security of the above two related schemes will be discussed in this section. Both of them are claimed that they can resist against various kinds of attacks and fulfill the desirable security requirements. However, we find that these two schemes are prone to smart card loss attack. Besides, they also suffer from two design flaws, including the impractical GWN search operation and no provision for perfect forward security.

#### 2.3.1. Smart Card Loss Attack

The smart card loss attack means that the password in the smart card can be guessed offline in the case where the smart card is lost or stolen. The authors of the above two schemes [28,29] have proved that their schemes are secure against this attack. The proofs assume that the identity of the user is unable to be guessed. However, since the identity of the user is a weak strength with low entropy, several articles [32,35,36] have proposed that the identity may be leaked when the smart card is lost or stolen. We now describe the details of this attack. 

For Lu et al.’s scheme [28], suppose that the adversary *A* has obtained the smart card of *U_i_*, and can extract secret information <*A_i_*,*B_i_*,*M_i_*,*b_i_*,*h*> from it, where *A_i_* = *h*(*h*(*ID_i_*)||*C_i_*), *B_i_* = *h*(*TID_i_*||*x*)⊕*C_i_*, *M_i_* = *h*(*ID_i_*||*x*)⊕*h*(*h*(*ID_i_*)⊕*C_i_*), *C_i_* = *h*(*PW_i_*||*b_i_*). Then *A* can successfully guess the *ID_i_* and *PW_i_* as below.

*Step 1*: *A* guesses a candidate pair *ID*’*_i_* and *PW*’*_i_*, and computes *C*’*_i_* = *h*(*PW*’*_i_*||*b_i_*), *A*’*_i_* = *h*(*h*(*ID*’*_i_*)||*C*’*_i_*).

*Step 2*: *A* checks whether *A*’*_i_* and *A_i_* stored in smart card are equivalent. If they are equal, *A* can obtain the correct *ID_i_* and *PW_i_* pair. Otherwise, *A* repeats the steps 1 and 2 until the correct *ID_i_* and *PW_i_* pair is obtained.

For Jung et al.’s scheme [29], the smart card stores <*N_i_*,*M_i_*,*b_i_*,*h*>, where *N_i_* = *h*(*ID_i_*||*C_i_*)⊕*v*, *M_i_* = *h*(*C_i_*||*v*), *v* = *h*(*x_i_*), *C_i_* = *h*(*PW_i_*||*b_i_*). Therefore, the process of launching smart card loss attack is similar, in many ways, to the process of attacking Lu et al.’s scheme. *A* can guess the correct *ID_i_* and *PW_i_* pair through checking whether *M_i_* = *h*(*h*(*PW*’*_i_*||*b_i_*)||*N_i_*⊕*h*(*ID*’*_i_*||*h*(*PW*’*_i_*||*b_i_*))) holds or not. 

Since the identity space |*D_ID_*| and the password space |*D_PW_*| are usually not more than 10^6^, the time required for *A* to complete this attack is linear [35]. As a result, Lu et al.’s scheme and Jung et al.’s scheme still fail to smart card loss attack. 

#### 2.3.2. Impractical GWN Search Operation

User anonymity is an important security feature of authentication scheme for WSNs, which consists of two properties, user identity-protection, and untraceability [36]. User identity-protection means that the adversary could not know the real identity of the user, and user untraceability guarantees that the adversary can neither determine who the user is nor distinguish whether two sessions are executed by the same user. To achieve user anonymity, the ‘dynamic ID technique’ is widely adopted in most schemes, so do Lu et al.’s scheme [28] and Jung et al.’s scheme [29]. In the two schemes, a user requires concealing his real identity into a dynamic identity. When the user wants to log in GWN, it is difficult for GWN to tell apart the real identity of the user. As a result, the GWN needs to search for every possible parameter or have a back-end channel to figure out the exact user, which is impractical [27]. The detailed of this operation will be described as follows. 

For Lu et al.’s scheme, the user sends a login message {*CT*_1_*,E_i_*} to GWN, where C*T*_1_ = *E_EK_*(*ID_i_*||*T*_1_||*TID_i_*||*r_i_*), *E_i_* = *h*(*h*(*ID_i_*||*x*)||*r_i_*||*T*_1_), *EK* = *h*(*TID_i_*||*x*) = *B_i_*⊕*C_i_*. After receiving the message {*CT*_1_*,E_i_*} from the user, the GWN decrypts *CT*_1_ by the symmetric key *EK* = *h*(*TID_i_*||*x*). Now, there is a problem that the GWN does not figure out exactly which *TID_i_* is the communicating user’s because all of the users’ *TID_i_* are stored in the GWN. The GWN has to perform an exhaustive search operation to obtain the exact user’s *TID_i_*. Let *L* is the size of user’s information table, *T_h_* is the execution time for hash operation and *T_E_* is the execution time for the decryption operation. The time complexity of the above operation is *O*(L*T_h_*T_E_). This is obviously impractical.

The similar problem can also be found in Jung et al.’s scheme. The user sends a login message {*DID_i_*,C*T*_1_,*T*_1_} to GWN, where C*T*_1_ = *E_EK_*(*DID_i_*||*R*_1_||*T*_1_), *EK* = *h*(*DID_i_*||*v*||*T*_1_), *DID_i_* = *h*(*ID_i_*||*R*_1_), *v = h*(*x_i_*). After receiving the message {*DID_i_*,C*T*_1_,*T*_1_} from the user, GWN decrypts *CT*_1_ by the symmetric key *EK* = *h*(*DID_i_*||*h*(*x_i_*)||*T*_1_), where *x_i_* is the shared symmetric key between the user and the GWN. Since all users’ shared symmetric keys are stored in the GWN, the GWN does not figure out exactly which one is the communicating user’s. It is obviously unrealistic for the GWN to perform an exhaustive search operation to obtain the exact user’s *x_i_*. Because the time complexity of the above operation is *O*(L*2T_h_*T_E_), where *L* is the size of user’s key table, *T_h_* is the execution time for hash operation and *T_E_* is the execution time for the decryption operation. 

#### 2.3.3. No Provision for Perfect Forward Secrecy

Perfect forward secrecy is one of the important security properties for authenticated key establishment protocols. A protocol is said to achieve the notion of perfect forward secrecy if the compromise of long-term keys does not compromise the previous session keys [33]. In the practical application, such as battlefield, the sensor node is unattended, which make it be dangerous in compromised by the adversary. Then the long-term key of the sensor node may be compromised and the previous session keys will be retrieved. Therefore, perfect forward secrecy should be considered for WSNs. However, none of the above two schemes [28,29] can provide perfect forward secrecy. 

For Lu et al.’s scheme, suppose the user’s long-term secret key *h*(*ID_i_*||*x*) and *h*(*TID_i_*||*x*) are compromised by the adversary *A*, and *A* has captured all the previous transmitted messages through the public communication channel. In this case, *A* is able to obtain all the previous message *CT*_4_. Thus, *A* can retrieve the past session keys through *r_k_*⊕*r_j_*||*r_i_*||*SID_j_*||*ID_GWN_*||*T*_2_||*T*_3_||*T*_4_ = *D_h_*_(*IDi*__⊕_*_x_*_)_(*CT*_4_), *SK* = *h*(*r_k_*⊕*r_i_*⊕*r_j_*||*T*_1_||*T*_2_||*T*_3_). Meanwhile, if the GWN’s long-term secret key *x* and the sensor node’s long-term secret key *K_GMN_S_* are compromised, the previous session keys will also be retrieved.

The similar problem can also be found in Jung et al.’s scheme, if the user’s long-term secret key *v* is compromised by *A*, and *A* has captured all the previous transmitted messages *DID_i_*, *CT*_3_*,* *T*_1_ through the public communication channel. Thus, *A* can retrieve the past session keys through *EK* = *h*(*DID_i_*||*v*||*T*_1_), *DID_i_*||*SID_j_*||*SK*||*R*_1_||*T*_4_ = *D_EK_*(*CT*_3_). Meanwhile, if the GWN’s long-term secret key *x_i_* and the sensor node’s long-term secret key *K_GWN_S_* are compromised, the previous session keys will also be retrieved. 

## 3. The Proposed Scheme

This section will describe each phase of the proposed anonymous authentication scheme for WSNs. It uses *PID* instead of the user’s real identity to protect user anonymity. In order to achieve the perfect forward secrecy, the transmitted messages in public channel are protected by the one-time hash chain technique. The back-end of GWN stores new *PID* and old *PID* during execution so as to resist against de-synchronization attack. The old *PID* will be set null until the GWN completes the authentication successfully. The proposed scheme consists of four phases: registration phase, authentication and key agreement phase, password update phase, and dynamically deploy sensor nodes phase. We will describe the detail in the upcoming subsection. 

### 3.1. Registration Phase

**Figure 4:**
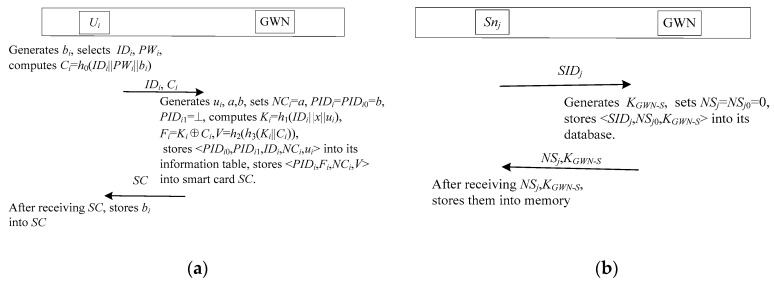
The registration phase. (**a**) The user registration phase; (**b**) The sensor node registration phase.

The registration phase includes user registration phase and sensor node registration. The details of these processes are described as follows. Figure 4 illustrates the registration phase for the proposed scheme.

#### 3.1.1. User Registration

When a user *U_i_* wants to access a sensor node *S_nj_*, he/she needs to register in GWN first. The GWN issues a smart card to *U_i_* as a response to the registration request. As shown in Figure 4a, the procedure of user registration is described as follows.

*Step 1*: A new user *U_i_* selects identity *ID_i_* and password *PW_i_*, generates a random number *b_i_*. Then *U_i_* computes *C_i_* = *h*_0_(*ID_i_*||*PW_i_*||*b_i_*), *U_i_* transmits {*ID_i_*,*C_i_*} to GWN through a secure channel.

*Step 2*: The GWN checks whether *ID_i_* exists in the user information table. If it exists, GWN rejects the registration request. Otherwise, GWN generates three random numbers *u_i_*,*a*,*b*, sets *NC_i_* = *a*, *PID_i_* = *PID_i_*_0_ = *b*, *PID_i_*_1_ = ⊥, and computes *K_i_* = *h*_1_(*ID_i_*||*x*||*u_i_*), *F_i_* = *K_i_*⊕*C_i_*,*V* = *h*_2_(*h*_3_(*K_i_*||*C_i_*)), where ⊥ denotes null. After that, GWN updates the user identity information table with the new entry {*PID_i_*_0_,*PID_i_*_1_,*ID_i_*,*NC_i_*,*u_i_*}, and stores {*PID_i_*,*F_i_*,*NC_i_*,*V*} into smart card *SC*. Finally, GWN sends *SC* to *U_i_* via a private channel.

*Step 3*: After receiving *SC* from GWN, *U_i_* stores *b_i_* into *SC*.

#### 3.1.2. Sensor Node Registration

When a new sensor node *S_nj_* is deployed, *S_nj_* is required to register in GWN. As shown in Figure 4b, the procedure of sensor node registration is described as follows.

*Step 1*: The new sensor node *S_nj_* selects identity *SID_j_* and transmits {*SID_j_*} to GWN through a secure channel.

*Step 2*: The GWN checks whether *SID_j_* exists in the sensor node information table. If it exists, the GWN rejects the registration request. Otherwise, the GWN generates a random number *K_GWN-S_*, and sets the initial sequence numbers *NS_j_* = *NS_j_*_0_ = 0. After that, GWN updates the sensor node information table with the new entry {*SID_j_*,*NS_j_*_0_,*K_GWN-S_*}, and sends {*NS_j_*,*K_GWN-S_* } to *S_nj_* via a private channel.

*Step 3*: After receiving *NS_j_*,*K_GWN-S_* from GWN, *S_nj_* stores them into its memory as secret.

### 3.2. Authentication and Key Agreement Phase

**Figure 5:**
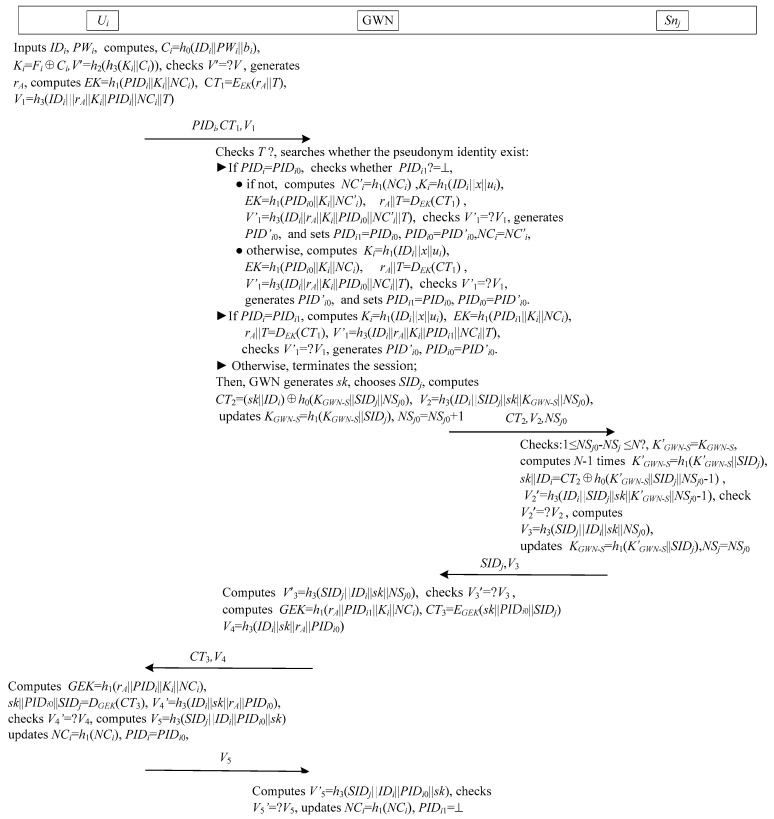
The authentication and key agreement phase.

When a user *U_i_* wants to gain access to WSNs, *U_i_* needs to achieve mutual authenticate with GWN and *S_nj_*. As shown in Figure 5, the process of mutual authentication is described as follows.

*Step 1*: *U_i_* inputs *ID_i_* and *PW_i_* into the smart card *SC*. *SC* computes *C_i_* = *h*_0_(*ID_i_*||*PW_i_*||*b_i_*), *K_i_* = *F_i_*⊕*C_i_, V*’ = *h*_2_(*h*_3_(*K_i_*||*C_i_*)), and compares *V*’ with the stored value *V*. If they are not equal, *SC* terminates the session. Otherwise, *SC* believes *U_i_* as a legitimate user. Next, *SC* generates a random number *r_A_*, and computes *EK* = *h*_1_(*PID_i_*||*K_i_*||*NC_i_*), *CT*_1_ = *E_EK_*(*r_A_*||*T*), *V*_1_ = *h*_3_(*ID_i_*||*r_A_*||*K_i_*||*PID_i_*||*NC_i_*||*T*), where *T* is the timestamp. Finally, *SC* sends the login request {*PID_i_*,*CT*_1_*,V*_1_} to GWN through the public channel.

*Step 2*: After receiving the login messages, GWN at first checks the timestamp *T*. Then GWN searches its back-end database to get each pair of the pseudonym identity (*PID_i_*_0_,*PID_i_*_1_) and operates as follows:

(1) GWN checks whether the pseudonym identity exists in the user information table.
  - If *PID_i_* = *PID_i_*_0_, it means that both the user’s and GWN’s pseudonym identity are updated in the previous session. Then GWN needs to verify whether the one-time hash chain value updates or not. GWN checks whether *PID_i_*_1_ = ⊥ holds.
    ✧ If the equation does not hold, it means that the GWN’s hash chain value does not update in the previous session. So, GWN computes *NC’_i_* = *h*_1_(*NC_i_*), *K_i_* = *h*_1_(*ID_i_*||*x*||*u_i_*), *EK* = *h*_1_(*PID_i_*_0_||*K_i_*||*NC’_i_*), *r_A_*||*T* = *D_EK_*(*CT*_1_), *V’*_1_ = *h*_3_(*ID_i_*||*r_A_*||*K_i_*||*PID_i_*_0_||*NC’_i_*||*T*). The GWN checks whether *V’*_1_ matches with the received *V*_1_. If it holds, GWN generates a random *PID’_i_*_0_, and sets *PID_i_*_1_ = *PID_i_*_0_, *PID_i_*_0_ = *PID’_i_*_0_, *NC_i_* = *NC’_i_*. Otherwise, GWN terminates the session.
    ✧ Otherwise, GWN computes *K_i_* = *h*_1_(*ID_i_*||*x*||*u_i_*), *EK* = *h*_1_(*PID_i_*_0_||*K_i_*||*NC_i_*), *r_A_*||*T* = *D_EK_*(*CT*_1_), *V’*_1_ = *h*_3_(*ID_i_*||*r_A_*||*K_i_*||*PID_i_*_0_||*NC_i_*||*T*). The GWN checks whether *V’*_1_ matches with the received *V*_1_. If it holds, GWN generates a random *PID’_i_*_0_, and sets *PID_i_*_1_ = *PID_i_*_0_, *PID_i_*_0_ = *PID’_i_*_0_. Otherwise, GWN terminates the session.
  - If *PID_i_* = *PID_i_*_1_, it means that the user’s pseudonym identity and hash chain are not updated in the previous session. GWN computes *K_i_* = *h*_1_(*ID_i_*||*x*||*u_i_*), *EK* = *h*_1_(*PID_i_*_1_||*K_i_*||*NC_i_*), *r_A_*||*T* = *D_EK_*(*CT*_1_), *V’*_1_ = *h*_3_(*ID_i_*||*r_A_*||*K_i_*||*PID_i_*_1_||*NC_i_*||*T*). GWN checks whether *V’*_1_ matches with the received *V*_1_. If it holds, GWN generates a random *PID’_i_*_0_, and sets *PID_i_*_0_ = *PID’_i_*_0_. Otherwise, GWN terminates the session.
  - If *PID_i_* ≠ *PID_i_*_0_, *PID_i_* ≠ *PID_i_*_1_, GWN terminates the session.
(2) GWN randomly generates a session key *sk* and chooses a specified sensor node *SID_j_*, and computes *CT*_2_ = (*sk*||*ID_i_*)⊕*h*_0_(*K_GWN-S_*||*SID_j_*||*NS_j_*_0_), *V*_2_ = *h*_3_(*ID_i_*||*SID_j_*||*sk*||*K_GWN-S_*||*NS_j_*_0_). Subsequently, GWN updates *K_GWN-S_* = *h*_1_(*K_GWN-S_*||*SID_j_*), *NS_j_*_0_ = *NS_j_*_0_ + 1.
(3) GWN sends {*CT*_2_,*V*_2_,*NS_j_*_0_} to the sensor node *S_nj_* that *U_i_* wants to interact with via the public channel.

*Step 3*: Upon receiving the messages {*CT*_2_,*V*_2_,*NS_j_*_0_} from GWM, *S_nj_* at first verifies whether 1≤*NS_j_*_0_*-NS_j_* ≤*N*, where *N* is a threshold, which sets according to specific requirements of applications. If it does not hold, *S_nj_* terminates the session. Otherwise, *S_nj_* set *K’_GWN-S_* = *K_GWN-S_*, and computes *N*-1 times *K’_GWN-S_* = *h*_1_(*K’_GWN-S_*||*SID_j_*), if *N*-1 = 0, there is no hash function operation. Next, *S_nj_* computes *sk*||*ID_i_* = *CT*_2_⊕*h*_0_(*K’_GWN-S_*||*SID_j_*||*NS_j_*_0_-1), *V*_2_’ = *h*_3_(*ID_i_*||*SID_j_*||*sk*||*K’_GWN-S_*||*NS_j_*_0_-1). Then, *S_nj_* checks whether *V’*_2_ matches with the received *V*_2_. If it holds, *S_nj_* computes *V*_3_ = *h*_3_(*SID_j_*||*ID_i_*||*sk*||*NS_j_*_0_), and updates *K_GWN-S_* = *h*_1_(*K’_GWN-S_*||*SID_j_*), *NS_j_* = *NS_j_*_0_. Otherwise, *S_nj_* terminates the session. Finally, *S_nj_* transmits {*SID_j_*, *V*_3_} to GWN.

*Step 4*: GWN first computes *V’*_3_ = *h*_3_(*SID_j_*||*ID_i_*||*sk*||*NS_j_*_0_), and checks whether *V’*_3_ matches with the received *V*_3_. If it holds, GWN computes *GEK* = *h*_1_(*r_A_*||*PID_i_*_1_||*K_i_*||*NC_i_*), *CT*_3_ = *E_GEK_*(*sk*||*PID_i_*_0_||*SID_j_*), *V*_4_ = *h*_3_(*ID_i_*||*sk*||*r_A_*||*PID_i_*_0_). Otherwise, GWN terminates the session. Finally, GWN transmits {*CT*_3_, *V*_4_} to *U_i_*. 

*Step 5*: *U_i_* computes *GEK* = *h*_1_(*r_A_*||*PID_i_*||*K_i_*||*NC_i_*), *sk*||*PID_i_*_0_||*SID_j_* = *D_GEK_*(*CT*_3_), *V*_4_*’* = *h*_3_(*ID_i_*||*sk*||*r_A_*||*PID_i_*_0_), and checks whether *V’*_4_ matches with the received *V*_4_. If it holds, *U_i_* computes *V*_5_ = *h*_3_(*SID_j_*||*ID_i_*||*PID_i_*_0_||*sk*), and updates *NC_i_* = *h*_1_(*NC_i_*), *PID_i_* = *PID_i_*_0_. Otherwise, *U_i_* terminates the session. Finally, *U_i_* sends {*V*_5_} to GWN. 

*Step 6*: After receiving the message *V*_5_ from *U_i_*, GWN computes *V’*_5_ = *h*_3_(*SID_j_*||*ID_i_*||*PID_i_*_0_||*sk*), and checks whether *V’*_5_ matches with the received *V*_5_. If it holds, GWN updates *NC_i_* = *h*_1_(*NC_i_*), *PID_i_*_1_ = ⊥. Otherwise, GWN fails to authenticate *U_i_*. 

Thus, the authentication key agreement among three-party is successful, and they establish the session key *sk* with each other as summarized in Figure 5.

### 3.3. Password Update Phase

When a user *U_i_* wants to update the password, he/she needs to run the following steps:

*Step 1*: *U_i_* inputs *ID_i_*, *PW_i_* into the smart card *SC*. *SC* computes *C_i_* = *h*_0_(*ID_i_*||*PW_i_*||*b_i_*), *K_i_* = *F_i_*⊕*C_i_*, *V*’ = *h*_2_(*h*_3_(*K_i_*||*C_i_*)), and checks whether *V*’ and *V* are equal. If not, *SC* fails to authenticate *U_i_*, and rejects the request of the password update. Otherwise *U_i_* inputs a new password PW*_i_. 

*Step 2*: SC computes C*_i_ = h_0_(ID_i_||PW*_i_||b_i_), F*_i_ = K_i_⊕C_i_⊕C*_i_, V* = h_2_(h_3_(K_i_||C*_i_)).

*Step 3*: Finally, F*_i_ and V* are stored in *SC* to replace *F_i_* and *V* respectively.

### 3.4. Dynamically Deploy Sensor Nodes Phase

When the system administrator deploys a new sensor node in the existing system, the deployed sensor node is required to apply to register in the GWN. The procedure of sensor node registration follows the steps described in Section 3.1.2.

## 4. Security Analysis of Our Scheme

In the section, we will discuss the security of our proposed scheme. First, the strand space model will be adopted to demonstrate the validity of our scheme. Second, we will demonstrate that our scheme provides mutual authentication and session key security using automated protocol verifier tool ProVerif. Finally, further security analysis illustrates the ability of the proposed scheme to resist various known attacks.

### 4.1. Authentication Proof Based on Strand Space Model

Strand space model [37,38] is a well-known formal analysis method to verify the security of cryptographic protocols. Before we prove the correctness of our proposed scheme using stand space mode, we will describe the basic notions as below. 

#### 4.1.1. The Basic Notion of Strand Space Model

According to [37,38], a stand space is a set *Σ* of stands with a trace mapping *tr*:*Σ* → (±*A*)*, which includes various protocol participant stands and penetrator strands. Where *A* is a set, the elements of which are the transmitted messages between principals. (±*A*)* is the set of finite sequences. The elements of *A* is denoted as terms *t*. *t*_1_ ⊏ *t* is defined as that *t*_1_ is a subterm of *t*. Due to the limitations of space, only the fundamental notations and lemmas in strand space model are enumerated here:

+*t*/-*t*: send/receive a term *t*.
<*s*,*i*>: a node of *s*, where *s* ∈ *Σ*, 1 ≤ *i* ≤ length(*tr*(*r*)). If *n* = <*s*,*i*>, then, index(*n*) = *i*, strand(*n*) = *s*, term(*n*) is the *i*th signed term in the trace of *s*, and uns_term(*n*) is the unsigned part of the *i*th signed term in the strand of *s*.
*n*_1_ → *n*_2_: it means that the node *n*_1_ sends a message and *n*_2_ receives the message.
*n*_1_ ⇒ *n*_2_: it means that *n*_1_ is an immediate causal predecessor of *n*_2_, *n*_1_ = <*s*,*i*> and *n*_2_ = <*s*,*i* + 1>.
*n*_1_ ⇒ ^+^ *n*_2_: it means that *n*_1_ is a precedence of *n*_2_, *n*_1_ = <*s*,*i*> and *n*_2_ = <*s*,*j*>, *i* < *j*.
*S*: a set of edges with respect to the causal relations →, ⇒ and ⇒ ^+^.
*n* ${≺}_{S}$ *n*’: it means that there are one or more edges in *S* leading from *n* to *n*’.
*n* ${\underline{≺}}_{S}$ *n*’: it means that there are zero or more edges in *S* leading from *n* to *n*’.
*T*: a set of atomic messages.
*K*: a set of cryptographic keys, which disjoints from *T*.
{*m*}_K_: it means that the message *m* is encrypted by the key *K*.

**Lemma** **1.** *Suppose C is a bundle. Then* ${\underline{≺}}_{C}$ *is a partial order, i.e., a reflexive, anti-symmetric, transitive relation. Every non-empty subset of the nodes in C has* ${\underline{≺}}_{C}$ *-minimal members.*

**Lemma** **2.** *Suppose C is a bundle. and suppose S is a set of nodes such that uns_term(m) = uns_term(m’) implies that m* ∈ *S iff m’* ∈ *S, for all nodes m,m’. If n is a* ${\underline{≺}}_{C}$*-minimal member of S, then the sign of n is positive*.

#### 4.1.2. Penetrator Strands

The following describes the abilities of an adversary, which are mainly characterized by the two factors, the one is the key set *K_p_* possessed by the adversary, the other is the capability of the adversary to generate new messages from messages he intercepts. The strands of the adversary/penetrator are as follows:

M. Text message: < + *t*>, the penetrator sends an atomic messages *t*, where *t* ∈ *T*.

F. Flushing: <-*g*>, the penetrator receives message *g*.

T. Tee: <-*g*, + *g*, + *g*>, the penetrator receives message *g* and forward it.

C. Concatenation: <-*g*,-*h*, + *gh*>, after receiving messages *g* and *h*, the penetrator joins them to get *gh*, then sends *gh*. 

S. Separation into components: <-*gh*, + *g*, + *h*>, upon receiving message *gh*, the penetrator sends message *g* and *h.*

K. Key: < + *K*>, the penetrator sends a key *K*, where *K* ∈ *K_P_*. 

E. Encryption: <-*K*,-*h*, + {*h*}*_K_*>, after receiving a key *K* and a message *h*, the penetrator encrypts *h* using *K*, and gets {*h*}*_K_*. Then, he sends {*h*}*_K_*.

D. Decryption: <-*K*^−1^,-{*h*}*_K_*, + *h*>, after receiving a private key *K*^−1^ and a ciphertext {*h*}*_K_*, the penetrator decrypts {*h*}*_K_* using *K*, and gets *h*. Then, he sends *h*.

H. Hash:<-*K*,-*M*, + *H*(*K*,*M*)>, after receiving a key *K* and a message *M*, the penetrator compute the hash value of *K*||*M*, and gets *H*{*K*||*M*}. Then, he sends *H*{*K*||*M*}.

#### 4.1.3. Authentication Proof Based on the Stand Space Model

The process of our proposed authentication scheme is as follows:

(1) *U_i_* → GWN: *PID_i_*, *CT*_1_, *V*_1_
(2) GWN → *S_nj_*: *CT*_2_, *V*_2_, *NS_j_*_0_
(3) *S_nj_* → GWN: *SID_j_*, *V*_3_
(4) GWN → *U_i_*: *CT*_3_, *V*_4_
(5) *U_i_* → GWN: *V*_5_

where *EK* = *h*_1_(*PID_i_*||*K_i_*||*NC_i_*), *CT*_1_ = *E_EK_*(*r_A_*||*T*), *V*_1_ = *h*_3_(*ID_i_*||*r_A_*||*K_i_*||*PID_i_*||*NC_i_*||*T*), *CT*_2_ = (*sk*||*ID_i_*)⊕*h*_0_(*K_GWN-S_*||*SID_j_*||*NS_j_*_0_), *V*_2_ = *h*_3_(*ID_i_*||*SID_j_*||*sk*||*K_GWN-S_*||*NS_j_*_0_), *V*_3_ = *h*_3_(*SID_j_*||*ID_i_*||*sk*||*NS_j_*_0_), *GEK* = *h*_1_(*r_A_*||*PID_i_*_1_||*K_i_*||*NC_i_*),*CT*_3_ = *E_GEK_*(*sk*||*PID_i_*_0_||*SID_j_*),*V*_4_ = *h*_3_(*ID_i_*||*sk*||*r_A_*||*PID_i_*_0_), *V*_5_ = *h*_3_(*SID_j_*||*ID_i_*||*PID_i_*_0_||*sk*).

The security goal of our proposed scheme is that the three participants should authenticate each other and share a secret key *sk*. In order to make the process of proof description clearer, we will refer to our proposed scheme using abbreviations LAAP.

**Figure 6:**
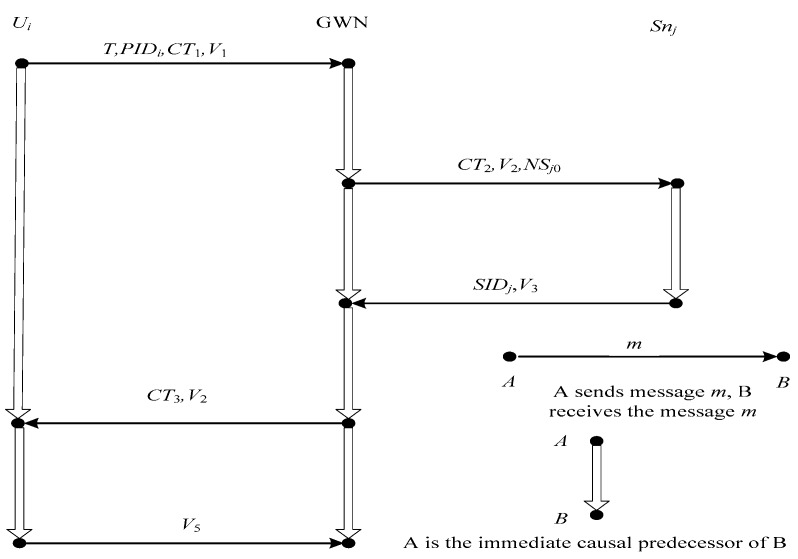
LAAP strands space model.

**Definition** **1.** *Let (Σ,P) be an infiltrated strand space. The strands space model is shown in Figure 6. (Σ,P) is a LAAP space if Σ have four kinds of strands.*

*(1)*  Penetrator strands s ∈ P.
*(2)*  ‘User strands’ with trace U[*ID_i_,SID_j_,T, r_A_,PID_i_,PID_i0_,K_i_,NC_i_,sk], defined to be < + {PID_i_, CT_1_, V_1_}, -{CT_3_, V_4_}, + {V_5_}>,* *where ID_i_, SID_j_ ∈ T_name_, r_A_, sk,PID_i_ ∈ T, K_i_,NC_i_ ∈ K, r_A_* ∉ *T_name_, r_A_* ∉ *K.*
*(3)*  ‘GWN strands’ with trace G[*ID_i_,SID_j_,T, r_A_,PID_i0_, PID_i1_,K_i_,NC_i_,sk,K_GWN-S_, NS_j0_],defined to be < −{PID_i_, CT_1_, V_1_}, + { CT_2_,V_2_,NS_j_ }, −{SID_j_, V_3_}, + { CT_3_,V_4_}, −{V_5_} >,* *where ID_i_, SID_j_ ∈ T_name_, r_A_, sk,PID_i_,PID_i0_,PID_i1_ ∈ T, K_i_,NC_i_,K_GWN-S_ ∈ K, sk* ∉ *T_name_, sk* ∉ *K.*
*(4)*  ‘Sensor node’ strands with trace Sn[ID_i_,SID_j_,sk,K_GWN-S_,NS_j0_],defined to be <−{CT_2_,V_2_,NS_j0_}, + {SID_j_, V_3_} >

If the user, the GWN, and the sensor node can achieve successful authentication with each other, our scheme is a secure authentication scheme. The details in the proof of our proposed scheme using strand space model is described in the Appendix A.

### 4.2. Formal Security Validation Using ProVerif

In this section, we will demonstrate that our scheme provides mutual authentication and session key security using automated protocol verifier tool ProVerif [39,40,41]. ProVerif is one of the widely used formal verification tools for cryptography protocols, which supports many cryptographic primitives, including symmetric and asymmetric cryptography, digital signatures, hash functions, Diffie-Hellman key agreements, and signature proofs of knowledge.

In order to analyze the security of our scheme by ProVerif, we define two public channels, c1 is the public channel between the user and the GWN and c2 is the public channel between the GWN and the sensor node. The proposed scheme is modeled as the parallel execution of three distinct processes: the user, the GWN and the sensor node. We have implemented the specifications in the latest version 1.96 of Proverif [42] for three processes. The implementation details of the proposed scheme are provided in the supplementary material available at [43]. 

ProVerif allows the verifier encrypts some free names using the secrecy session key, and verifies the security of session key by test the secrecy of that free names [41]. As shown in Figure 3, we use four names secretA, secretB, secretC and secretD for secrecy queries to analyze the secrecy of session key *sk*. To verify mutual authentication, we declare eight events:

event beginUGparam(host), event endUGparam(host),
event beginGUparam(host), event endGUparam(host),
event beginGSparam(host), event endGSparam(host),
event beginSGparam(host), event endSGparam(host).

Intuitively, if one participant believes he has completed the scheme with another participant and hence executes the event endXXparam(host), where XX denotes UG, GU, GS, or SG. The results show that our scheme can achieve mutual authentication and session key security. We describe the results of the code as below:

➢ Query not attacker(secretA[]), not attacker(secretB[]),not attacker(secretC[]), not attacker(secretD[])
  ✧ RESULT not attacker(secretA[]), not attacker(secretB[]), not attacker(secretC[]), not attacker(secretD[]) are true.
  ✧ The result means that the adversary has not trace to reconstruct secretA, secretB, secretC, secretD. Hence, the session key *sk* is secure to resist cracking.
➢ Query inj-event(endGUparam(GWN)) ═> inj-event(beginGUparam(GWN))
  ✧ RESULT inj-event(endGUparam(GWN)) = = > inj-event(beginGUparam(GWN)) is true.
  ✧ This result means that the execution of the event beginGUparam(GWN) is preceded by the execution of the event endGUparam(GWN). Hence, the authentication of the user to the GWN holds.
➢ Query inj-event(endUGparam(user)) ═> inj-event(beginUGparam(user))
  ✧ RESULT inj-event(endUGparam(user)) ═> inj-event(beginUGparam(user)) is true.
  ✧ This result means that the execution of the event beginUGparam(user) is preceded by the execution of the event endUGparam(user). Hence, the authentication of the GWN to the user holds.
➢ Query inj-event(endGSparam(GWN)) ═> inj-event(beginGSparam(GWN))
  ✧ RESULT inj-event(endGSparam(GWN)) ═> inj-event(beginGSparam(GWN)) is true.
  ✧ This result means that the execution of the event beginGSparam(GWN) is preceded by the execution of the event endGSparam(GWN). Hence, the authentication of the sensor node to the GWN holds.
➢ Query inj-event(endSGparam(SN)) ═> inj-event(beginSGparam(SN))
  ✧ RESULT inj-event(endSGparam(SN)) ═> inj-event(beginSGparam(SN)) is true.
  ✧ This result means that the execution of the event beginSGparam(GWN) is preceded by the execution of the event endSGparam(GWN). Hence, the authentication of the GWN to the sensor node holds.

### 4.3. Further Security Analysis of Our Scheme

In this section, the ability of the proposed scheme to resist various known attacks will be analyzed. 

#### 4.3.1. Resistance to De-synchronization Attack

**Figure 7:**
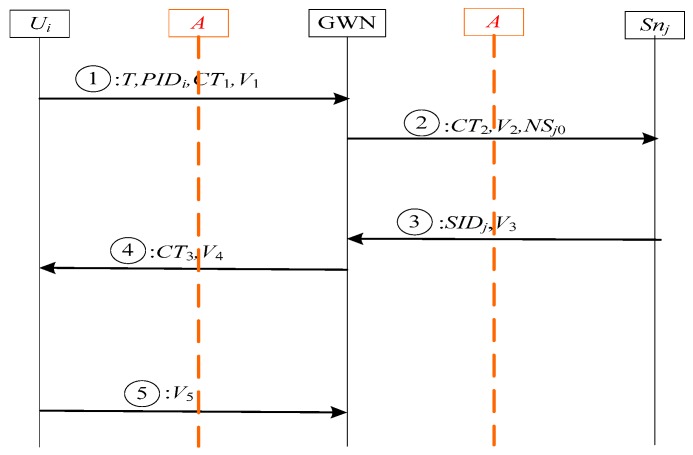
The de-synchronization on our proposed scheme.

Our scheme employs *PID* and one-time hash chain techniques to provide user anonymity and perfect forward secrecy. Hence, it needs an additional synchronization method to maintain the consistency of several one-time values among the user, the GWN, and the sensor node. In the proposed scheme, the consistencies of *PID* and hash chain value will be ensured by using two pseudonym identities < *PID_i_*_0_,*PID_i_*_1_ > for the communication between the user and the GWN. For the communication between the GWN and the sensor node, we use the serial number to resist de-synchronization attack. Since the hash function is one way, we let the initiator updates the hash chain value at first. As a result, even if the adversary blocked the message, the hash chain value of the GWN and the sensor node can re-synchronize. In order to make our analysis clearer, a brief framework of our scheme is shown in Figure 7. 

The adversary can launch the following malicious scenarios:

Scenario 1: If the adversary blocks the ① message flow, obviously, this attack will not work because all the participants have not even started updating. So, this scenario will be omitted.

Scenario 2: If the ② message flow is blocked by the adversary, the communication will be jammed. For the communication between the *U_i_* and the GWN, this scenario is the same as scenario 4. For the communication between the GWN and *S_nj_*, the hash chain values of two participants will not match each other. This attack does not cause our scheme completely unusable because we use serial number *NS_j_*_0_ and *NS_j_* to record the number of hash chain updated, where *NS_j_*_0_ is the serial number of GWN side, *NS_j_* is the serial number of *S_nj_* side. When the GWN sends the ② message flow, the value of hash chain and *NS_j_*_0_ in GWN side must be updated. The *S_nj_* receives the ② message {*CT*_2_,*V*_2_,*NS_j_*_0_}, he/she can synchronize the one-time hash chain value through performing *NS_j_*_0_*-NS_j_* time hash functions. Therefore, this scenario will cause asynchronous between the GWN and the *S_nj_*, but it will not have any impact on the future session.

Scenario 3: If the adversary blocks the ③ message flow, obviously, this attack will not work between the GWN and the *S_nj_* because the two participants have updated their hash chain values, and the hash chain values are equal to each other. For the communication between the *U_i_* and the GWN, this scenario is the same as scenario 4. Therefore, this scenario will be omitted.

Scenario 4: If the ④ message flow is blocked by the adversary, this attack will not work between the GWN and the *S_nj_* because both of them have updated hash chain values. But the communication between the *U_i_* and the GWN will be jammed. In this scenario, since both the hash chain values in two participants are not changed, only the synchronization of pseudonym identities are required to consider. The value of *PID_i_*_0_ in the GWN side has been a new pseudonym identity, while the value of *PID_i_* in the *U_i_* side does not change. Fortunately, the old pseudonym identity is stored in *PID_i_*_1_ in the GWN side, that is *PID_i_*_1_ = *PID_i_*. So, when the next session is initiated by the *U_i_* using unchanged *PID_i_*, the GWN is still able to recognize it and continues to complete the authentication. Therefore, this scenario will cause pseudonym identity asynchronous between the *U_i_* and the GWN, but it will not have any impact on the future session. 

Scenario 5: If the ⑤ message flow is blocked by the adversary, like scenario 4, this attack will not work between the GWN and the *S_nj_*. However, for the communication between the *U_i_* and the GWN, it will be jammed. Since the pseudonym identities values of two participants have updated, it means *PID_i_*_0_ = *PID_i_*, we only need to worry about the synchronization of two participants’ hash chain values. In this scenario, the hash chain value in the *U_i_* side is updated, while the value hash chain in the GWN side is unchanged. When *U_i_* using changed hash chain value initiates a new session, the GWN will update its hash chain value through checking whether the value of *PID_i_*_1_ is non-null or not. Therefore, even if this scenario will cause hash chain value asynchronous between the *U_i_* and the GWN, the two pseudonym identities will make the hash chain values synchronize again. As a result, our scheme can resist de-synchronization attack through the above analyses.

#### 4.3.2. Mutual Authentication

According to the proofs of Proposition A1–Proposition A4 and the formal validation using ProVerif, it is infeasible for an adversary to forge a legitimate user’s or GWN’s or sensor node’s authentication message. Thus, the user, the GWN, and the sensor node can successfully authenticate each other.

#### 4.3.3. User Anonymity

To protect user’s identity, the proposed scheme employs pseudonym identity as a transmitted message instead of user’s real identity. The pseudonym identity is randomly generated and changes after completing each session. Thus, the pseudonym identity is different for every session. Moreover, it is almost impossible for an adversary to get the user’s real identity from transmitted messages. Therefore, our scheme is able to support user anonymity and untraceability. 

#### 4.3.4. Perfect Forward Secrecy

In the proposed scheme, suppose the adversary has obtained the long-term keys of two participants, that are *K*_i_, *NC_i_*, and *K_GWN-S_*, he/she still cannot get the session key *sk*. The reason is that after each successful session, the keys *NC_i_* and *K_GWN-S_* will be updated by one-way hash function, that is *NC’_i_* = *h*_1_(*NC_i_*), *K’_GWN-S_* = *h*_1_(*K_GWN-S_*||*SID_j_*). Because the hash function is one way, the adversary cannot obtain *NC_i_* and *K_GWN-S_* from *NC’_i_* and *K’_GWN-S_*. Therefore, our scheme can provide perfect forward secrecy.

#### 4.3.5. Resistance to Smart Card Loss Attack

Suppose the adversary steals the user’s smart card and obtains the data {*PID_i_*,*F_i_*,*NC_i_*,*V,b_i_*}, where *K_i_* = *h*_1_(*ID_i_*||*x*||*u_i_*), *F_i_* = *K_i_*⊕*C_i_*,*V* = *h*_2_(*h*_3_(*K_i_*||*C_i_*)), *C_i_* = *h*_0_(*ID_i_*||*PW_i_*||*b_i_*). The adversary cannot guess the correct password, because there exist |D_ID_|*|D_PW_|/1024 candidates of the password, where |D_ID_|is the space of the identity and |D_PW_| is the space of the password. This method is called ‘fuzzy verifier’ [23,44,45], which prevents the adversary from obtaining the exacting correct password. Therefore, our proposed scheme can resist smart card loss attack.

#### 4.3.6. Resistance Known Session-Specific Temporary Information Attack

In the proposed scheme, suppose the adversary gets the ephemeral random number *r_A_*, he still cannot obtain information of session key *sk*. The reason is that the adversary has no way to compute the long-term key *K_i_*, one-time hash chain values *NC_i_* and *K_GWN-S_*. Moreover, transmitted messages in the public channel are unhelpful to compute *sk*. Therefore, the proposed scheme has the ability to prevent the session-specific temporary information attack.

#### 4.3.7. Resistance to Stolen Verifier Table Attack

In our scheme, no any password-verifier table of the user is stored in the GWN side. Therefore, our scheme can resist stolen verifier table attack.

#### 4.3.8. Resistance to User Impersonation Attack

In our scheme, in order to forge a user, the adversary has to generate a valid value {*T,PID_i_,CT*_1_*,V*_1_}. However, it is infeasible because the adversary does not know the secret keys *K_i_* and *NC_i_*. Therefore, our proposed scheme can resist against user impersonation attack.

#### 4.3.9. Resistance to Sensor Node Spoofing Attack

Proposition A1–Proposition A4 and the formal validation using ProVerif show that the adversary cannot forge a legitimate user’s or sensor node’s authentication message without the secret keys *K_i_*, *NC_i_* or *K_GWN_S_*. In the proposed scheme, the sensor node only has his own secret value and does not know the secret values of other sensor nodes or users. Therefore, he cannot spoof any user or other sensor nodes.

#### 4.3.10. Resistance to Replay Attack

The proposed scheme uses timestamp, nonce and serial number to prevent the replay attack. For the communication between the user and the GWN, the first message flow includes a current timestamp *T*, and other message flow employs challenge-response mechanism to resist reply attack. For the communication between the GWN and the sensor node, the serial number is used in every message flow, which is updated after each successful authentication session. As a result, when the user and the sensor node accept each other, it must be the current session, not previous session. Therefore, our proposed scheme can avoid the replay attack.

#### 4.3.11. Resistance to Man-in-the-middle Attack

In the proposed scheme, the transmitted messages are protected by the secret values *K_i_*, *NC_i_* and *K_GWN_S_*, anyone without them cannot forge legal authentication messages. Therefore, our scheme can resist man-in-the-middle attack.

#### 4.3.12. Resistance to Wrong Password Login/Update Attack 

In the proposed scheme, the password verification information *V* = *h*_2_(*h*_3_(*K_i_*||*C_i_*)) is stored in the mobile device, which is designed to check the correctness of password. If the user inputs wrong password *PW*’*_i_*, the verification data *V* and *V’* = *h*_2_(*h*_3_(*F_i_*⊕*h*_0_(*ID_i_*||*PW’_i_*||*b_i_*)||*h*_0_(*ID_i_*||*PW’_i_*||*b_i_*))) will not be equal. Therefore, our scheme can quickly detect unauthorized login and password update.

### 4.4. Security Comparisons

The security features of our proposed scheme with the two prior related schemes [28,29] will be compared in this section. The results of the comparison are listed in Table 2. 

From Table 2, it can be concluded that the proposed scheme is the only one who can resist against various kinds of known attacks and fulfill the desirable security features. Therefore, our scheme has better security than the previously related schemes.

## 5. Performance Analysis

This section will compare the communication and communication costs of our proposed scheme with the two prior related schemes [28,29]. Since the registration phase and password update phase are not used frequently, we only concentrate on comparing authentication phase. 

### 5.1. Computation Analysis

For efficiency analysis, we compare the computation costs of our scheme with the two prior related schemes [28,29]. To facilitate analysis, we use the following notations to measure computation costs.

- *T_h_*: the time complexity of the general hash function.
- *T_E/D_*: the time complexity of general symmetric-key encryption/decryption algorithm.

As pointed out in [46,47], the running time of a one-way hash function operation, and symmetric-key encryption/decryption operation are 0.00032s and 0.0056s respectively. Thus, we have *T_h_* ≈ 0.00032s, *T_E/D_* ≈ 0.0056s. The results of the computation complexity comparisons of our scheme and two related schemes are summarized in Table 3. It shows that our scheme is as efficient as the most efficient one of these prior related schemes at sensor nodes. Although the computation cost for the user and the GWN of our proposed scheme is higher than that of Jung et al.’s scheme, it should be toleratable because our proposed scheme provides higher security, and resists most well-known attacks.

### 5.2. Communication Analysis

In this section, we compare the communication cost of our proposed scheme with the two prior related schemes [28,29]. To achieve convincing comparisons, we assume that the bit length of identity (*ID_i_*,*SID_j_*,*ID_GWN_*), password (*PW_i_*), pseudonym identity (*PID_i_*,*PID_i_*_0_,*PID_i_*_1_), timestamp (*T*,*T*_1_,*T*_2_,*T*_3_,*T*_4_), serial number (*NS_j_*_0_, *NS_j_*), random number (*r_A_*,*sk*), hash (*h*,*h*_1_,*h*_3_) output and hash (*h*_0_) output are 64, 64, 64, 160, 64, 256, 160 and 320 bits, the block length of the symmetric encryption is 128 bits, respectively. Since the bit length of ciphertext using the symmetric encryption is the multiples of 128 bits, the bit length of *CT*_1_ and *CT*_3_ are 512 and 384 bits, respectively. Table 4 shows the communication cost comparison among our scheme and the prior related schemes. In our scheme, the message {*PID_i_,CT*_1_*,V*_1_}, {*CT*_2_*,V*_2_*,NS_j_*_0_}, {*SID_j_*,*V*_3_}, {*CT*_3_,*V*_4_} and {*V*_5_} require (64 + 512 + 160) = 736, (320 + 160 + 64) = 544, (160 + 64) = 224, (384 + 160) = 544 and 160 bits, respectively. Adding the five values, the total communication cost of our scheme is 2208 bits. 

For Lu et al.’s scheme [28], the message {*CT*_1_*,E_i_*}, {*CT*_2_*,C_i_*}, {*CT*_3_,*I_i_*} and {*CT*_4_,*V_i_*}require ((64 + 160 + 64 + 256) + 160) = 128 × 5 + 160 = 800, ((256 + 64 + 160 + 160) + 160) = 128 × 5 + 160 = 800, ((256 + 256 + 160) + 160) = 128 × 6 + 160 = 928, and ((256 + 256 + 64 + 64 + 160 × 3) + 160) = 128 × 9 + 160 = 1312 bits, respectively. Adding the four values, the total communication cost of Lu et al.’s scheme is 3840 bits. 

For Jung et al.’s scheme [29], the message {*DID_i_,CT*_1_*,T*_1_}, {*CT*_2_*,DID_i_,B_i_,T*_2_}, {*C_i_,T*_3_} and {*CT*_3_,*T*_4_} require (64 + (64 + 256 + 160) + 160) = 64 + 128 × 4 + 160 = 736, (256 + 64 + 160 + 160) = 640, (160 + 160) = 320, and ((64 + 64 + 160 + 256 + 160) + 160) = 128 × 6 + 160 = 928 bits, respectively. Adding the four values, the total communication cost of Jung et al.’s scheme is 2624 bits.

Using the above similar approach, the total communication cost of the other related schemes can be computed in Table 4. From comparison in Table 4, it can be concluded that the proposed scheme has the least communication cost among the above schemes.

## 6. Conclusions

In this paper, we propose a lightweight anonymous authentication protocol for WSNs based on a one-time hash chain and pseudonym identity. The proposed scheme can provide mutual authentication, user anonymity, perfect forward secrecy, etc. Besides, it is resilient to various kinds of known attacks, such as de-synchronization attack, and known session-specific temporary information attack. Formal security analysis and simulations are also conducted by ProVerif to demonstrate that our scheme is secure against active and passive attacks. Furthermore, the proposed scheme only uses symmetric key encryption/decryption and hash functions. It is very suitable for the resource constrained sensor nodes. 

## Figures and Tables

**Table 1:** Notations.

Notation	Descriptions
*U_i_*	The user
*S_nj_*	The sensor node
GWN	The gateway node
*SC*	The smart card
*ID_i_*,*PW_i_*	Unique identity and password of *U_i_*
*SID_j_*	Unique identity of *S_nj_*
*ID_GWN_*	Unique identity of GWN
*PID*	Pseudonym identity
*PID_i_*	Pseudonym identity of *U_i_* in the user side
*TID_i_*	A random number of *U_i_* generated in the GWN
*x*	The secret key of GWN
*x_i_*	The shared secret key between GWN and *U_i_*
*K_GWN_S_*	The shared secret key between GWN and *Sn_j_*
*b_i_*	A random number generate by *U_i_*
*SK*	The session key
*E_K_*,*D_K_*	Encryption/Decryption using the symmetric key *K*
*h*,*h_0_*,*h*_1_,*h*_3_	One-way hash function
*h*_2_	One-way hash function, *h*_2_:{0,1}*→{0,1,...,1023}
*T*,*T*_1_,*T*_2_,*T*_3_,*T*_4_	Current timestamp
*||*	String concatenation operation
⊕	XOR operation

**Table 2:** Security features comparisons of our scheme and the two related schemes.

Features	Lu et al. [28]	Jung et al. [29]	Ours
Resistance to de-synchronization attack	√	√	√
Mutual authentication	√	√	√
User anonymity	√	√	√
Perfect forward security	×	×	√
Smart card loss attack	×	×	√
Resistance to known session-specific temporary information attack	√	√	√
Resistance to stolen verifier table attack	√	√	√
Resistance to user impersonation attack	√	√	√
Resistance to sensor node spoofing attack	√	√	√
Resistance to replay attack	√	√	√
Resistance to man-in-the-middle attack	√	√	√
Resistance to wrong password login/update attack	√	√	√

**Table 3:** Computation complexity comparisons of our scheme and the two related schemes.

Schemes	Users	GWN	Sensor Node	Total
Lu et al. [28]	7*T_h_ +* 2*T_E/D_* ≈ 0.01344s	8*T_h_ +* 4*T_E/D_* ≈ 0.02496s	4*T_h_ +* 2*T_E/D_* ≈ 0.01248s	19*T_h_ +* 8*T_E/D_* ≈ 0.05088s
Jung et al. [29]	5*T_h_ +* 2*T_E/D_* ≈ 0.0128s	5*T_h_ +* 2*T_E/D_* ≈ 0.0128s	4*T_h_* ≈ 0.00128s	13*T_h_ +* 4*T_E/D_* ≈ 0.02688s
Ours	9*T_h_ +* 2*T_E/D_* ≈ 0.01408s	11*T_h_ +* 2*T_E/D_* ≈ 0.01472s	4*T_h_* ≈ 0.00128s	25*T_h_ +* 4*T_E/D_* ≈ 0.03008s

**Table 4:** Communication cost comparisons of our scheme and the two related schemes.

Schemes	Number of Message Required	Number of Bits Required
Lu et al. [28]	4 Messages	3840
Jung et al. [29]	4 Messages	2624
Ours	5 Messages	2208

**Figure A1:**
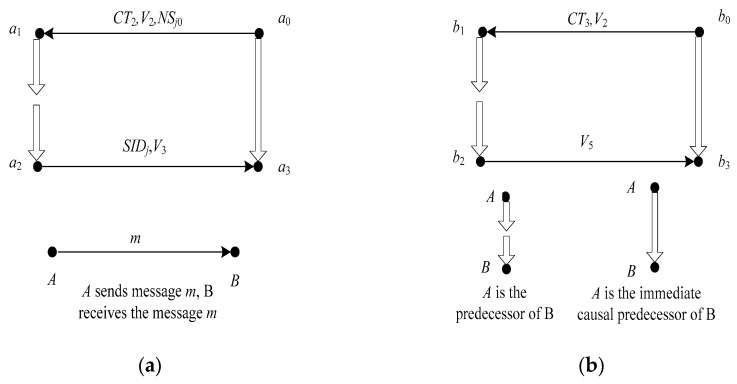
The nodes of a GWN’s strand. (**a**) The nodes *a*_0_ ≺ *a*_1_ ≺ *a*_2_ ≺ *a*_3_; (**b**) The nodes *b*_0_ ≺ *b*_1_ ≺ *b*_2_ ≺ *b*_3_.

## Appendix A. The Details in the Proof of Our Proposed Scheme Using Strand Space Model

**Proposition** **A1.** Suppose:

(1) *Σ* is a LAAP space and C is a bundle containing a GWN’s strand *s* with trace G[*ID_i_*,*SID_j_*,*T*, *r_A_*,*PID_i_*_0_, *PID_i_*_1_,*K_i_*,*NC_i_*,*sk*,*K_GWN-S_*, *NS_j_*_0_];
(2) *EK* ∉ *K_P_*, *K_GWN-S_* ∉ *K_P_*, where *EK* = *h*_1_(*PID_i_*||*K_i_*||*NC_i_*); and
(3) *r_A_* ≠ *PID_i_*_0_ ≠ *sk* ≠ *PID_i_* ≠ *PID_i_*_1_, *PID_i_*_0_ and *sk* are uniquely originating in *Σ*.

then *C* contains a user’s strand with trace U[*ID_i_*,*SID_j_*,*T*,*r_A_*,*PID_i_*,*PID_i_*_0_,*K_i_*,*NC_i_*,*sk*], and a sensor node’s strand with Sn[*ID_i_*,*SID_j_*,*sk*,*K_GWN-S_*,*NS_j_*_0_]. We will prove Proposition A1 using the following lemmas. For the sake of convenience, we will refer to <*s*,2> (that is the second node + {*CT*_2_,*V*_2_,*NS_j_*} of *s*) as *a*_0_, and to its term(that is term(*a*_0_)) as *u*_0_. We will refer to <*s*,3> (that is the third node + {*SID_j_*, *V*_3_} of *s*) as *a*_3_, and to its term(that is term(*a*_3_)) as *u*_3_. We will refer to other nodes similarly. The node <*s*,4> is denoted as *b*_0_, and term(*b*_0_) = *v*_0_. The node <*s*,5> is denoted as *b*_3_, and term(*b*_3_) = *v*_3_. As shown in Figure A1, we will use four additional nodes *a*_1_,*a*_2_,*b*_1_,*b*_2_ during the course of the proof, such that *a*_0_ ≺ *a*_1_ ≺ *a*_2_ ≺ *a*_3_, *b*_0_ ≺ *b*_1_ ≺ *b*_2_ ≺ *b*_3_. First, we will prove *C* contains a sensor node’s strand with Sn[*ID_i_*,*SID_j_*,*sk*,*K_GWN-S_*,*NS_j_*_0_].

**Lemma** **A1.** sk originates at a_0_.

**Proof.** By the assumptions, *sk* ⊏ *u*_0_, and the sign of *a*_0_ is positive. According to the definition of originates, we only require verifying that *sk* is not the subterm of a node *a*’, where *a*’ is the precedence node of *a*_0_. Since on the same strand, the precedence node of *a*_0_ is <*s*,1>, uns_term(<*s*,1>) = -{*PID_i_*,*CT*_1_*,V*_1_}, where *EK* = *h*_1_(*PID_i_*||*K_i_*||*NC_i_*), *CT*_1_ = *E_EK_*(*r_A_*||*T*), *V*_1_ = *h*_3_(*ID_i_*||*r_A_*||*K_i_*||*PID_i_*||*NC_i_*||*T*). We need to check that *sk* ≠ *r_A_*, *sk* ≠ *PID_i_,* which is a hypothesis, *sk* ≠ *K_i_*, *sk* ≠ *NC_i_*, *sk* ≠ *SID_j_*, *sk* ≠ *ID_i_*, which follows from the stipulation in Definition 1 Clause 3 that *sk* ∉ *T_name_*, *sk* ∉ *K*. ☐

**Lemma** **A2.** *The set S = {a ∈ C: sk* ⊏ *term(a)* ∧ *u_0_!* ⊏ *term(a)} has a minimal node a_2_, u_0_!* ⊏ *term(a) denotes as u_0_ that is the subterm of term(a). The node a_2_ is regular and the sign of a_2_ is positive*.

**Proof.** Because *a*_3_ ∈ *C*, *sk* ∈ *C*, *u*_0_! ⊏ *u*_3_, *S* is non-empty. Therefore, *S* has at least a minimal element *a*_2_ by Lemma 1, and the sign of the node *a*_2_ is positive by Lemma 2. Whether *a*_2_ lie on a penetrator stand *p*? We will check it through the form of the trace of *p*. ☐

M. Text message: < + *t*>, where *t* ∈ *T*. If this stand contains the node *a*_2_, which means that *sk* originates on this strand. Accord to Lemma A1, *sk* originates at *a*_0_. Obviously, it is a contradictory assumption. Therefore, M strand cannot contain the node *a*_2_.

F. Flushing: <-*g*>, since this strand lacks any positive nodes, F strand cannot contain the node *a*_2_.

T. Tee: <-*g*, + *g*, + *g*>, the penetrator receives message *g* and forward it. T strand cannot contain the node *a*_2_ because *a*_2_ is a minimal element.

C. Concatenation: <-*g*,-*h*, + *gh*>, after receiving messages *g* and *h*, the penetrator joins them to get *gh*, then sends *gh*. C strand cannot contain the node *a*_2_ because *a*_2_ is a minimal element.

K. Key: < + *K*>, the penetrator sends a key *K*,where *K* ∈ *K_P_*. K strand cannot contain the node *a*_2_ because *sk*! ⊏ *K*.

E. Encryption: <-*K*,-*h*, + {*h*}*_K_*>, after receiving a key *K* and a message *h*, the penetrator encrypts *h* using *K*, and gets {*h*}*_K_*. Then, he sends {*h*}*_K_*. Suppose the node + {*h*}*_K_* ∈ *S*, since *sk* ⊏ {*h*}*_K_* ∧ *u*_0_! ⊏ {*h*}*_K_*, thus *sk* ⊏ *h,* ∧ *u*_0_! ⊏ *h*. E strand cannot contain the node *a*_2_ because the positive node is not in *S*.

D. Decryption: <-*K*^−1^,-{*h*}*_K_*, + *h*>, after receiving a private key *K*^−1^ and a ciphertext {*h*}*_K_*, the penetrator decrypts {*h*}*_K_* using *K*, and gets *h*. Then, he sends *h*. If the node + *h* is a minimal element *a*_2_, then *u*_0_! ⊏ *h* ∧ *u*_0_ ⊏ {*h*}*_K_*. According to the assumption of free encryption, *h* = *sk*||*ID_i_*, and *K* = *h*_0_(*K_GWN-S_*||*SID_j_*||*NS_j_*_0_). So, there must exist a penetrator strand which can send *K*. Obviously, it is contradictory because *K_GWN-S_* ∉ *K_P_*. Therefore, D strand cannot contain the node *a*_2_.

H. Hash:<-*K*,-*M*, + *H*(*K*,*M*)>, after receiving a key *K* and a message *M*, the penetrator compute the hash value of *K*||*M*, and gets *H*{*K*||*M*}. Then, he sends *H*{*K*||*M*}. According to the assumption, *K* = *sk*, and *M* = *SID_j_*||*ID_i_*||*NS_j_*_0_. So, there must exist a penetrator strand which can send *K*. Obviously, it is contradictory because *sk* is secret, and any penetrator without *K_GWN-S_* can know it. Therefore, H strand cannot contain the node *a*_2_.

S. Separation into components: <−*gh*, + *g*, + *h*>, upon receiving message *gh*, the penetrator sends message *g* and *h.* Suppose, the unsigned term minimal element uns_term(*a*_2_) = *g*(there is a similar case if uns_term(*a*_2_) = *h*)*.* Since *a*_2_ ∈ *S*, then *sk* ⊏ *g* ∧ *u*_0_! ⊏ *g*. There must have *u*_0_ ⊏ *gh*, or the term of the minimal element in *S* is *gh*. Because, *u*_0_! ⊏ *g*, *u*_0_ ⊏ *gh*, there must have *u*_0_ ⊏ *h*. Let a set *T* = {*m* ∈ *C*: *m* ≺ *a*_2_ ∧ *gh* ⊏ term(*m*)}. Every element of *T* is a penetrator node because there is no regular node contains a subterm *gh*, where *u*_0_ ⊏ *h*. T is non-empty because of the first node of S strand -*gh* ∈ *C.* Therefore, *T* has at least a minimal element *m* by Lemma 1, and the sign of the node *m* is positive by Lemma 2. We will check what kind of stand *m* can lie on.

Obviously, similar to the above analysis, M,F,T,C,K,S,E,D cannot contains the minimal element of *T*. Therefore, the node *a*_2_ does not lie on *p* but must lie on a regular node.

**Lemma** **A3.** *The precedence node of a_2_ is on the same regular strand. Let it as a_1_, and term(a_1_) = {CT_2_,V_2_,NS_j_}*.

**Proof.** Because *sk* originates at *a*_0_ and *sk* are uniquely originating in *Σ*, *sk* must not originate at *a*_2_. According to Lemma A2, *a*_2_ lie on a regular node, there must be a node preceding *a*_1_ on the same strand such that *sk* ⊏ term(*a*_1_). Since *u*_0_! ⊏ term(*a*_2_), and *a*_2_ is the minimal element, *u*_0_ ⊏ term(*a*_1_). Besides, there is no regular node contains *u*_0_. Therefore, term(*a*_1_) = {*CT*_2_,*V*_2_,*NS_j_*}. ☐

**Lemma** **A4.** The strand containing a_1_ and a_2_ is a sensor node strand, and is contained in C.

**Proof.** According to Lemma A3, term(*a*_1_) = {*CT*_2_,*V*_2_,*NS_j_*}, and it is the precedence node of *a*_2_. Because *a*_2_ is the minimal element, and it is a positive node. Beside, *a*_0_ is a positive node, and term(*a*_0_) = {*CT*_2_,*V*_2_,*NS_j_*}. Therefor, *a*_1_ = -{*CT*_2_,*V*_2_,*NS_j_*}, *a*_2_ = + {*SID_j_*,*V*_3_}, it must be an sensor node strand. ☐

Lemma A3 and Lemma A4 shows *C* contains a sensor node’s strand with Sn[*ID_i_*,*SID_j_*,*sk*,*K_GWN-S_*,*NS_j_*_0_]. Moreover, we can prove that *C* contains a user node’s strand with U[*ID_i_*,*SID_j_*,*r_A_*,*PID_i_*,*PID_i_*_0_,*K_i_*,*NC_i_*,*sk*] using the above similar proof methods.

**Proposition** **A2.** If Σ is an LAAP space, C is a bundle, and sk are uniquely originating in Σ, then there are at most one sensor node strand t_1_ with trace Sn[ID_i_,SID_j_,sk,K_GWN-S_,NS_j0_] for any GWN, sensor node and sk.

**Proof.** If any sensor node strand *t*_1_ has trace Sn[*ID_i_*,*SID_j_*,*sk*,*K_GWN-S_*,*NS_j_*_0_] any user, GWN, and *sk*, the <*t*_1_,2> is positive, *sk* ⊏ term<*t*_1_,2>, and *sk* is the challenge information of GWN. Hence, if *sk* originates uniquely in *Σ*, there can be at most one such *t*_1_. ☐

**Proposition** **A3.** If Σ is an LAAP space, C is a bundle, and r_A_ is uniquely originating in Σ, then there are at most one user strand t_2_ a with trace U[ID_i_,SID_j_,r_A_,PID_i_,PID_i0_,K_i_,NC_i_,sk] for any user, GWN, and sk.

**Proof.** If any user strand *t*_2_ has trace U[*ID_i_*,*SID_j_*,*r_A_*,*PID_i_*,*PID_i_*_0_,*K_i_*,*NC_i_*,*sk*] any user, GWN, and *sk*, the <*t*_2_,1> is positive, *r_A_* ⊏ term<*t*_2_, 1>, and *r_A_* cannot possibly occur earlier on *t*_2_. Therefore, *r_A_* originates at node <*t*_2_, 1>. Hence, if *r_A_* originates uniquely in *Σ*, there can be at most one such *t*_2_. ☐

**Proposition** **A4.** Suppose:

(1) *Σ* is a LAAP space and *C* is a bundle containing a user’s strand *s* with trace U[*ID_i_*,*SID_j_*,*r_A_*,*PID_i_*,*PID_i_*_0_,*K_i_*,*NC_i_*,*sk*] and a sensor node’s strand *t* with Sn[*ID_i_*,*SID_j_*,*sk*,*K_GWN-S_*,*NS_j_*_0_];
(2) G*EK* ∉ *K_P_*, *K_GWN-S_* ∉ *K_P_*, where *GEK* = *h*_1_(*r_A_*||*PID_i_*_1_||*K_i_*||*NC_i_*); and
(3) *r_A_* ≠ *PID_i_*_0_ ≠ *sk* ≠ *PID_i_* ≠ *PID_i_*_1_, *PID_i_*_0_ and *sk* are uniquely originating in *Σ*.

then *C* contains a GWN’s strand with trace G[*ID_i_*,*SID_j_*,*r_A_*,*PID_i_*_0_, *PID_i_*_1_,*K_i_*,*NC_i_*,*sk*,*K_GWN-S_*, *NS_j_*_0_].

**Proof.** In order to prove Proposition A4, we can refer to the GWN’s strand as a responder’s strand of the user or a initiator’s stand of the sensor node. As the responder’s strand and as the initiator’s stand, the processes of proofs are similar with Proposition A1. Take the case of GWN as an responder’s strand, we only consider the set *S* = {*a* ∈ *C*: {*V*_4_} ⊏ term(*a*)} is non-empty. Because <*s*_2_,2> is the element of *S*, *S* has at least a minimal element *a*_0_. If *a*_0_ is on the regular strand *g*, it is easy to prove *g* ∈ G[*ID_i_*,*SID_j_*,*r_A_*,*PID_i_*_0_, *PID_i_*_1_,*K_i_*,*NC_i_*,*sk*,*K_GWN-S_*, *NS_j_*_0_]. Otherwise, *g* should be on the H strand with trace<-*GEK*,-*r_A_*||*sk*||*PID*_0_, + {*V*_4_}>. Obviously, it is contradictory because *GEK* ∉ *K_P_*. Therefore, *C* contains a GWN’s strand with trace G[*ID_i_*,*SID_j_*,*r_A_*,*PID_i_*_0_, *PID_i_*_1_,*K_i_*,*NC_i_*,*sk*,*K_GWN-S_*, *NS_j_*_0_]. Proposition A1–Proposition A4 show that our scheme is a secure mutual authentication scheme among the user, the GWN and the sensor node. ☐
